# Supplementary material for: Therapeutic genetic variation revealed in diverse Hsp104 homologs
Source: eLife. 2020 Dec 15;9:e57457. doi: 10.7554/eLife.57457 (PMC7785292; doi:10.7554/eLife.57457)
Supplement: Supplementary file 1. — The primary sequence of each Hsp104 homolog is provided. [file elife-57457-supp1.docx]

**Supplementary File 1. Amino acid sequences of Hsp104 homologs.**

| **Species** |  |
| --- | --- |
| *Galdieria sulphuraria* | MNLDNLTDKAQEAIQASHELALENGHSQLTPLHLAAALFTEDHGLASSVATKAKADPVNVRRELQKAVIRLPSQDPPPTTVPPSQSFLKVIRDAQSLRKKQGDTHLAVDHLLIALCDDKDVIACFSSADLTKHALEEAVKSVRGNRKVDSKAADSTYDALNQYAQDFVALAEEGKLDPVIGRDDEIRRVIRVLCRRRKNNPVLIGDPGVGKTAIVEGLAQRIVRGDVPENLNCRLYALDMGALVAGAKYRGEFEERLKAVLREVKEGEGKIILFIDELHLVLGAGKSDGAMDAANLLKPMLARGELRCIGATTLEEYRKYVEKDAAFERRFQQVFVSEPSVPDTVSILRGLKERYEVHHGVRILDSALVAAAKLSARYITNRFLPDKAIDLVDEACANVRMQLDSQPEVIDTLEHRKLQLEIEIAALEKEKDEASRARLAAVKEELDNVNEKLRPLKARFESERGKMNELKDMMTKLDALKIKLADAERRRDTVQAADLRYYAIPEIEERIRSLKNEIDKEKETDAMETDEESGKLLSDVVGYEQIADVVSRWTGIPTTKLSQSDAERLLSLSASLHQRIIGQDEAVDAVAAAVLRSRAGVSRPTQPLGSFLFLGPTGVGKTELAKALAAELFDDEKHVVRIDCSEYMEQHSVSRLIGAPPGYVGYEEGGQLTEAVLRRPYNVVLFDEVEKAHRNVMNVLLQVLDDGRLTDNQGRTIDFTNTVIILTSNLGAQFLMNIGSKGPAELSEGSDHEGTPVVSIPKEATIDNRTREAVMREVKLHFRPEFLNRLDDIVIFKPLALDELRQIVRLQLDQVAKRLEERDITVSMDNRAADYILREAYDPSFGARPIRRYLEKHVATELSIRLIKGLLTNHSHVQVTRATEGSGLAFQVTPKKRSATDMGNGSVYNKQGIVEEPDEEDM |
| *Monosiga brevicollis* | MAINPNEFTDKVNKTLFEAQNFAIQEGHSQVEPAHVAVILFEDPEGMAKRVVQRAGAALQPVQAALRSLLQRMPRQEPAPLEASLSSDTRRLLQSAAKLQKKNNEAHLAVDHLLGALVQDKQILAKLAESGLAKNHFEETLKRVKGTTTADSKSAEENYDALSKYGVDLVQQAADGKLDPVLGRDEEIRRVIQILARRIKSNPCLVGPPGVGKSAIVEGLAQRIMLGDVPETLKGKLISLDMGALIAGAKYRGEFEERLKAVLEEIKQSEGRIILFVDEVHNVLGAGKTEGSMDAANLLKPLLARGELRMIGATTEDEYRKYVEKDSAFERRFQVVQVREPSVPDTVSILRGLKERYEAHHGVRIADAALVAAAQLSHRYIQGRFLPDKAIDLIDEACANARVQLDSRPEEIDQLERRRLQLQVEATALEKEKDQASKLRLKDVRKELANIEEQLQPLLMKFEMERGRVDELRDLQEKLDSLRSKAQRAERQGDLATAADLKYYAIPDCERRIQQLTLEDEERSAQRSGMDEQEDAPMLSEEVGPEQITDIIARWTGIPVTKLNQSQRERLLALAERIKSRVIGQDHAVDAVAEAVLRSRAGLSRPSQPTGSFLFLGTTGVGKTELAKALAAELFDDDKHIVRIDMSEYMESHAVSRLIGSPPGYVGYEQGGQLTEAVRRRPYNVVLFDEVEKAHPQVLNVLLQVLDDGVLTDGQGRHVDFTNTVIVLTSNIGAHDLLNADVVNGAIDPETEAKVRRQVQQHFRPEFLNRLDEVVMFKPLGQRDLRKICRNMVDLINQRLVDRDIALLVSDDACDLVLDEAYNPAYGARPVRRYVEKHMVTEISRLVLSGELVNHSTVHIDTTKRPDGGRDLSYQVHHSKRPKISEDSA |
| *Chlamydomonas reinhardtii* | MSFDTKKATEKVNNVLGEAINLAKEDKHAALTPTHLAVVLFEEPHGLAKVAATKVAGEEVWRSAIRVLRKRLTKLPKVDPAPESVSPGRELSKVLTAAAKLQKDRGDAFLGTDTLLTAVINAAEVSEALGEAGISKAQLETALSEVRQAAGGGPINSETADANFDALAKYGTDLTANAARADPVIGRDDEIRRVVRVLCRRTKNNPVLIGEPGVGKTAIVEGLAQRIVKNDVPETLQGVRLISLDMGSLVAGAKYRGEFEERLKAVLNEVAQQQGKVVLFIDELHLVLGAGKSGDGAMDAANLLKPMLARGELRCIGATTLGEYREHIEKDAAFERRFQQVLVKEPSVPDTIAILRGIKDRYETHHGVHITDRALVVAAELSDRYITTRFLPDKAIDLVDEACANMRVQLDSKPEQLDALERQRQRLQVEAAALAKEKDALSKARAKEVGKELAALEEALRPLQMKYAQEKARLEELRRLGQKRDEILVNIQIAEQHGNLARIADLRYGALPDVEDRIKQVRAAAPSDAMLSEEVGTEEIAVVVSRWTGIPVNRLKQTERDKLLSLRSELQQRVVGQDAAVAAVADAVLRSRAGLAARGRGSSFLFLGPTGVGKTELAKALAQLLFDDEKMMIRIDMGEYMEKHSVSRLIGAPPGYVGHEQGGQLTEAVRRRPYSVVLFDEVEKAHAEVFNVLLSILDDGRVTDSKGRTVNFANTVIILTSNLGAEALLHAAHDVLAHPGKAAAGQDPYKAARESVLAAVRRFFRPEFLNRLDDIVVFEPLRPEQLVDIARLMGNELAARLTPRNITLTFTEPALQFAVSHAYDPAYGARPLRRWMEQKVVTQLSRMVVGGDLPDNSNVEVGLAEGGRDFDYRVWPKPAAEAGGGNGSGAATEALLKKARMYSVEHPDEGEDSMDEGDDVGAMRD |
| *Populus*  *euphratica* | MDPGKFTHKTNEALATAHELTVGAGHAQITPLHLAVALISDPSGIMRQAVANAGDGENTAQAAERVFNQVLKKLPSQSPPPDEVPPSTSLIKVIRRSQALQKSRGDSYLAVDQMILGLLEDSQIRDLFKEVGVSASTVKSEVEKLRGKEGKKVENASGDTNFQALKTYGRDLVEGAGKLDPVIGRDEEIRRVVRILSRRTKNNPVLIGEPGVGKTAVAEGLAQRIVRGDVPSNLADVRLIALDMGALVAGAKYRGEFEERLKAVLKEVEEAEGKVILFIDEIHLVLGAGRTEGSMDAANLFKPMLARGQLRCIGATTLEEYRKYVEKDAAFERRFQQVYVAEPSVPDTISILRGLKEKYEGHHGVRIQDRALVIAAQLSSRYITGRHLPDKAIDLVDEACANVRVQLDSQPEEIDSLERKRMQLEVELHALEKEKDKASKARLAEVVKELDDLRDKLQPLLMKYKKEKERIDEIRRLKQKREEILFSIQEAERRYDLARVADLRYGALEEVEAAIARLEGSTTDENLMLTETVGPEHIAEVVSRWTGIPFTRLGQNEKERLIGLADRLHHRVVGQDQAVTAVAEAVLRSRAGLGRPQQPTGSFLFLGPTGVGKTELAKALAEQLFDDENQLVRIDMSEYMEQHSVARLIGAPPGYVGHEEGGQLTEAVRRRPYSVVLFDEVEKAHISVFNTLLQVLDDGRLTDGQGRTVDFRNTVIIMTSNLGAEHLLSGLLGKCSMQVARDRVMQEVRKQFRPELLNRLDEIVVFDPLSHDQLRKVARLQMKDVASRLAERGIALAVTDAALDYILAESYDPVYGARPIRRWLERKVVTELSRMLVREEIDENSTVYIDAGPDGQNLVYRVEKNGGLVNAATGQKTDVLIQIPKAPRDDAAQKVKKMKIQEIVDNDDDDEMIE |
| *Salpingoeca*  *rosetta* | MALNPNEWTEKVQEMYLEAKNVAINNKNAYMDPIHFAVALFEDEGGLPQRVVQKSGASLDAVEGAMRSLLKAIPQQDPAPVDVSTSHKALRFLQNAQKKQKKNDEAHLAIDHLLLALVQEKDILQALAGCGLAKDRFEEIVKKIKGTTRANTKTAESTYDALGKYGVDLVQRAADGKLDPVIGRDEEIRRVIQILARRTKNNPVLVGPPGTGKTAIVEGLAQRILNGDVPETLKARLVSLDMGALIAGAKYRGEFEERLKSVLDEVKQAEGSIILFVDEIHTVLGAGKTEGSMDAANLLKPMLARGELRMIGATTLDEYRKHVEKDAAFERRFQMVHVSEPSVPDTVSILRGLKERYEAHHGVRIQDAALVTAAQLADRYITQRFLPDKAIDLVDEACAKTRVQLDSRPEEIDALERRKLQLEVEATALGKEKDKMSKQRLKEVKKQLADIEEQLGPLKMKFEMERGRVDEMRELQEKLDNLRNKVQRAERAGDLSTAADLKYYAIPDCEKRLKQLIEEQEKRQQEQQSMDVSDEDKPMLSEEVGPDQVTEIVARWTGIPVNKLSQSQRERLLTLEEKIEQRVVGQTAAVKAVCEAVLRSRAGLSRPNQPVGSFLFLGPTGVGKTELAKALAMELFDDDKHIVRVDMSEYMESHSVARLIGSPPGYVGYEEGGQLTEAVRRRPYNLILLDEVEKAHKDVLNVLLQLLDDGILTDGMGRTVDFTNTVVVLTSNIGAPILLSGKVDPETGDLDENTRTQVMHEVQSYFRPEFLNRLDDVIMFKPLQKHALRTICRNMVEQINERLADRDITLDCTDAACDVILLNSFHPQYGARPVRRYIEKQVVTAMSKKMLSNQVPNGSRVTIDGDKQRQELTYSVQAAKRPRMMMDSTSEE |
| *Thielavia*  *terrestris* | MEFTDRAKKALEDAMALAEQYSHSQLVPVHLAVALLDPLPDQSKDQQNAPPGTTSSLFRQVVERANGDPQLFDRALKKSLVRLPSQEPPPESVSMAPSLNTVLRKAMELQKVQKDTYIAVDHLITALSEDPSIQVALKEANIPKSKLVQDAVTAIRGTRRVDSRNADTEQENENLSKFTIDMTAMAREGKIDPVIGREEEIRRVIRILSRRTKNNPVLIGEPGVGKTTIVEGLAQRIVNADVPDNLAACKLLSLDVGALVAGSKYRGEFEERMKGVLKEIQESKETIVLFVDEIHLLMGAGATGEGGMDAANLLKPMLARGQLHCIGATTLGEYRKYIEKDAAFERRFQQVLVKEPSIPETISILRGLKEKYEVHHGVTIADAAIVAAANLAARYLTSRRLPDSAVDLIDEAAAAVRVARESQPEIIDSLERRLRQLKIEIHALSREKDDASKARLAQAKQDAQNVEEELRPLREKYERERQRGKDIQEAKLKLENLRVKAEDASRMGDHGRAADLQYYAIPEQEQVIKRLEKEKAAADAALNANGADVGGSMVTDVVGPEQINEIVARWTGIPVTRLKTSEKEKLLHMERALSKIVVGQKEAVQSVSNAIRLQRSGLSNPNQPPSFLFCGPSGTGKTLLTKALAEFLFDDAKAMIRFDMSEYQERHSLSRMIGAPPGYVGHDAGGQLTEALRRKPFSILLFDEVEKAAKEVLTVLLQLMDDGRITDGQGRVVDAKNCIVVMTSNLGAEYLSRPNGKDGQIDPTTKELVMNTLRNYFLPEFLNRISSIVIFNRLTRREIRKIVDLRIAEIQRRLYDNDRNVQIAVSEAAKDKLGAAGYSPAYGARPLQRLLEKEVLNRLAILILRGNIRDGEVAHVDLVDGKVTVQPNHPDSEGEDEEMMVDEDEALEEIAPDSMDEDDIYSG |
| *Thermomyces*  *lanuginosus* | MSSAQFTDRANKALLDSNQLAEQYSHTQILPVHLAVSLLNPPRDDEREGAEGASVPLFKQVVERAHGDPQLLERSLMKMLVRQPSQDPPPEHVSVSPALAKVIRSATELSKMQKDSFVAIDHLICALAQDSQIQRALADANIPNVKLIDNAVQQIRGTKRVDSKTADSEEDHENLKRFTVDMTALAREGKIDPVIGREEEIRRVIRILTRRTKNNPVLIGEPGVGKTTIVEGLARRIVNADVPANLAHCKLLSLDVGSLVAGSKYRGEFEERMKGVLKEIEESRDMVVLFVDEIHLLMGAGTSGEGGMDAANLLKPMLARGQLHCIGATTLAEYRKYIEKDQAFERRFQQVFVKEPSVSETISILRGLKEKYEVHHGVNILDAAIVASAELAARYLTARRLPDSAVDLIDEAAAAVRVTRESEPEALDNLERRLRQLQIEIHALEREKDPASKSRLEAAKQEAANVTEELRPLREKYEQEKQRSKAIQDAKVKLDALKVKRDEALRSGDTATAADLEYYAIPETQSLIQKLEAERAKADAEERARAHENGTETLVPDAVGPDQINEIVARWTGIPVTRLKTSEKDRLLQMEKHLGKIVVGQKEAVQTVSNAIRLQRSGLANPNAPPSFLFCGPSGTGKTLLTKALAEFLFDDPKAMVRFDMSEYQERHSLSRMIGAPPGYVGHDAGGQLTEHLRRRPFSILLFDEVEKAAKEVLTVLLQLMDDGRITDGQGRVVDAKNCIVVMTSNLGAEYLSRPTGKNGRIEPQTRELVMGALRDYFLPEFLNRISSIVIFNRLTKREIRQIVELRLDEVQRRLEANGRDVKIECADEVKDYLGEAGYSPAYGARPLSRLIEKEVLNKLAILLLRGNIRDGEVAHCVLKDGRVDVIPNHPSDEMEDDEDMIDPNEALEQVEDTSADMDLYE |
| *Saccharomyces*  *cerevisiae* | MNDQTQFTERALTILTLAQKLASDHQHPQLQPIHILAAFIETPEDGSVPYLQNLIEKGRYDYDLFKKVVNRNLVRIPQQQPAPAEITPSYALGKVLQDAAKIQKQQKDSFIAQDHILFALFNDSSIQQIFKEAQVDIEAIKQQALELRGNTRIDSRGADTNTPLEYLSKYAIDMTEQARQGKLDPVIGREEEIRSTIRVLARRIKSNPCLIGEPGIGKTAIIEGVAQRIIDDDVPTILQGAKLFSLDLAALTAGAKYKGDFEERFKGVLKEIEESKTLIVLFIDEIHMLMGNGKDDAANILKPALSRGQLKVIGATTNNEYRSIVEKDGAFERRFQKIEVAEPSVRQTVAILRGLQPKYEIHHGVRILDSALVTAAQLAKRYLPYRRLPDSALDLVDISCAGVAVARDSKPEELDSKERQLQLIQVEIKALERDEDADSTTKDRLKLARQKEASLQEELEPLRQRYNEEKHGHEELTQAKKKLDELENKALDAERRYDTATAADLRYFAIPDIKKQIEKLEDQVAEEERRAGANSMIQNVVDSDTISETAARLTGIPVKKLSESENEKLIHMERDLSSEVVGQMDAIKAVSNAVRLSRSGLANPRQPASFLFLGLSGSGKTELAKKVAGFLFNDEDMMIRVDCSELSEKYAVSKLLGTTAGYVGYDEGGFLTNQLQYKPYSVLLFDEVEKAHPDVLTVMLQMLDDGRITSGQGKTIDCSNCIVIMTSNLGAEFINSQQGSKIQESTKNLVMGAVRQHFRPEFLNRISSIVIFNKLSRKAIHKIVDIRLKEIEERFEQNDKHYKLNLTQEAKDFLAKYGYSDDMGARPLNRLIQNEILNKLALRILKNEIKDKETVNVVLKKGKSRDENVPEEAEECLEVLPNHEATIGADTLGDDDNEDSMEIDDDLD |
| *Dictyostelium*  *discoideum* | MSFNPEEFTDKTNTILLRTQELAREKSNVQLAPIHLAVTLLNDEDNLAKSIFEKAGGDVPKIDAGFKRLLAKQPVQNPVPPEISPNSLFLQVLRSAARHQKNNGDSHLAVDHLILGLLDDRDILSVLGDAGATKEQVIQAVKEIRGNKKITSKTAESTYEALSKYGYDLVSQAQEGKLDPVIGRDEEIRRVIRVLSRRTKNNPVLIGEPGVGKTAVVEGLAQRIVRGDIPDNLNARVIALDMGALIAGAKYRGDFEERLKAVLKEVKDSNGGIILFIDEIHLVLGAGKTDGAMDAANLLKPMLARGELRCIGATTLDEYRQYVEKDPAFERRFQQVFVNEPTVNDTISILRGLKERYETHHGVRITDNALVVAAQLSHRYITNRFLPDKAIDLVDEACANTRVQLNSQPEAIDNLERRRLQLEVESAALEKEEDDASKQRLKAVQEELNQIKDELQPLEAKYQKERSRVDKIRELRKKLEDIKVKLSDAERRYDTSAAADYRYFVIPDLEKQIEVCDKERKENKKDAMVSEVVTPEQIADVVSRWTGIPVSKLSQTEKQRLLSLADHLHNRVVGQDEAVDAVADAVLRSKSGLARENQPLGSFLFLGPTGVGKTELAKALALELFDDESHMVRIDMSEYMEQHSVSRLIGAPPGYVGYDQGGQLTEAVRRRPYSVVLFDEVEKAHQQVWNVLLQVLDEGRLTDGQGRTVDFSNVVIIMTSNLGSQYILGEQANKEGGNNSLSQACKDKVIDEVRKHFRPEFLNRLDDIIVFTPLSKENLHSIITLQLRSVEKRLEDQNMSLKISNDALDSIINAAYDPIFGGRPLKRYIEKNIVTELSKLILGGKLKENQGVVVNEKDHHLNFDIIDLKQQQQRPASPTKKQKTIK |
| ClpG_GI_, from *Pseudomonas aeruginosa* | MAQELCAICHERPAVARVSLVQNGQRRELALCELHYRQLMRQQRMRSPLESLFGGGSPFDEIFSGFGEQSPVTPVRAREPEAVDIAEYFSKQTTEYLQRAAQVAAEFGKREVDTEHLLYALADADVVQAVLKQFGLSPADLKQYIEANAVRGASKGEASEDMTISPRVKSALQHAFALSRELGHSYVGPEHLLLGLAAVPDSFAGTLLKKYGLTEQALRQKAVKVVGKGAEDGRVDGPSNTPQLDKFSRDLTRLAREGKLDPVIGRSKEVETTIEVLARRKKNNPVLIGEPGVGKTAIVEGLAQRMVQGEVPEVLRDKRLVELNINAMVAGAKYRGEFEERLKQVMDELQAAQSEIILFIDEVHTIVGAGQGGGEGGLDVANVLKPAMARGEMNLIGATTLNEYQKYIEKDAALERRFQPVFVPEPTVEQTISILRGLRDKLEGHHKVTIRDEAFVAAAELSDRYIGNRFLPDKAIDLIDQAAARVRIASTSRPAEIQELEAELAQLKREQDYAASRKWYDEAKVFEKRIQERKEHLEQITERWQQTQGSKTEEVRVEDIAEIISRLTGIPVTELTAEEREKLLQMEERLHQRVIGQQEAITAVSDAVRLARAGLRQGSRPIATFLFLGPTGVGKTELAKALAEVVFGDEDAMIRIDMSEYMERHAVSRLIGAPPGYVGYDEGGQLTERVRRRPYSVILLDEIEKAHADVNNILLQVFDDGRLTDGKGRVVDFTNTIIIATSNLGSELIMKNAQAGEFAQPPEKLKRELMTTLRGHFRPEFLNRLDEVIVFESLSKAQIEDIVRLQLERVKRAAHAQDIYLHIDDSLVGHLAEEAYQPEFGARELKRQIRQQLETRLATAMLKGEVKEGETVTFFYDAKDGVGYRKGAAPKPAARKKSGAGETPKGRATAARKPAAKKGAAAKGKADKPKAK |
| ClpB, from *Escherichia coli* | MRLDRLTNKFQLALADAQSLALGHDNQFIEPLHLMSALLNQEGGSVSPLLTSAGINAGQLRTDINQALNRLPQVEGTGGDVQPSQDLVRVLNLCDKLAQKRGDNFISSELFVLAALESRGTLADILKAAGATTANITQAIEQMRGGESVNDQGAEDQRQALKKYTIDLTERAEQGKLDPVIGRDEEIRRTIQVLQRRTKNNPVLIGEPGVGKTAIVEGLAQRIINGEVPEGLKGRRVLALDMGALVAGAKYRGEFEERLKGVLNDLAKQEGNVILFIDELHTMVGAGKADGAMDAGNMLKPALARGELHCVGATTLDEYRQYIEKDAALERRFQKVFVAEPSVEDTIAILRGLKERYELHHHVQITDPAIVAAATLSHRYIADRQLPDKAIDLIDEAASSIRMQIDSKPEELDRLDRRIIQLKLEQQALMKESDEASKKRLDMLNEELSDKERQYSELEEEWKAEKASLSGTQTIKAELEQAKIAIEQARRVGDLARMSELQYGKIPELEKQLEAATQLEGKTMRLLRNKVTDAEIAEVLARWTGIPVSRMMESEREKLLRMEQELHHRVIGQNEAVDAVSNAIRRSRAGLADPNRPIGSFLFLGPTGVGKTELCKALANFMFDSDEAMVRIDMSEFMEKHSVSRLVGAPPGYVGYEEGGYLTEAVRRRPYSVILLDEVEKAHPDVFNILLQVLDDGRLTDGQGRTVDFRNTVVIMTSNLGSDLIQERFGELDYAHMKELVLGVVSHNFRPEFINRIDEVVVFHPLGEQHIASIAQIQLKRLYKRLEERGYEIHISDEALKLLSENGYDPVYGARPLKRAIQQQIENPLAQQILSGELVPGKVIRLEVNEDRIVAVQ |
| *Chaetomium*  *thermophilum* | MNSKMEFTDRAKKALEDAMALAEQYQHLQLQPVHLAVALLDPTPDPSKDQSIAPGTTSTLFRQVVERAHGDAQAFDRALKKKLVRLPSQDPPPDQVSMSAGCSNVLRKANELQKVQKDSYIAVDHLIAALAEDHAIQEALKEANIPKPKLIQDAIQAIRGNKRVDSRNADTEQENENLSKFCIDMTAMAREGKIDPVIGREEEIRRVIRILSRRTKNNPVLIGEPGVGKTTIVEGLAQRIVNADVPDNLAACKLLSLDVGALVAGSKYRGEFEERMKGVLKEIQESKETIILFVDEIHLLMGAGSSGEGGMDAANLLKPMLARGQLHCIGATTLAEYRKYIEKDAAFERRFQQVLVKEPSISETISILRGLKEKYEVHHGVNIADAAIVAAANLAARYLTSRRLPDSAVDLIDEAAAAVRVARESQPEIIDSLERRLRQLKIEIHALSREKDEASKARLAQAKQDAQNVEEELRPLREKYERERQRGKAIQEAKMKLEALRVKAEDASRMGDHSRAADLQYYAIPEQEAIIKRLEAEKAAADAALNANGADVGGSMITDVVGPDQINEIVARWTGIPVTRLKTSEKEKLLHMEQALSKIVVGQKEAVQSVSNAIRLQRSGLSNPNQPPSFLFCGPSGTGKTLLTKALAEFLFDDPKSMIRFDMSEYQERHSLSRMIGAPPGYVGHDAGGQLTEALRRRPFSILLFDEVEKAAKEVLTVLLQLMDDGRITDGQGRVVDAKNCIVVMTSNLGAEYLSRANNGKDGKIDPTTRELVMNTLRNYFLPEFLNRISSVVIFNRLTRREIRKIVDLRIAEIQKRLTDNDRNVIIKVSEEAKDKLGAQGYSPVYGARPLQRLLEKEVLNRLAILILRGQIREGEVAHVELVDGKVQVLPNHPDSEPEDVDVDMDSDDAVDEVAPDSMDEDIYND |
| *Lachancea*  *thermotolerans* | MNDETQFTERALTILTLAQKLAQDHQHAQLQPVHILGAFVETPEDGSIPYLQNLIEKARYDYDTFRRTVNKHVVRIPQQNPAPAQVTPSYATGQVLQEAMKIQKQQKDSFVAQDHILFALFKDSTIQQIFKEAQVDVEAVKQQALELRGNQKIDSRGADTSSSLEYLSKYAIDMTEQARMGKLDPVIGREEEIRSTIRVLARRIKSNPCLIGEPGIGKTAIIEGVAQRIIDDDVPSILFGSKLFSLDLAALTAGAKYKGDYEERLKGVLKEVEESKTLIILFIDEIHMLMGNGKDDAANILKPALSRGHLKVIGATTNNEYRSIVEKDGAFERRFQKIDVQEPTTRQTVAILRGLQQKYEIHHGVRILDSALVTASQLAKRYLPYRRLPDSALDLVDISCAGVAVARDSKPEELDSKERALQLLQVEIKALERDEEADPTTKERLQQARQREASLQEELEPLRQRYNEERKGHEELTKAKKKLEELENKAADAERRYDTATAADLRYFAIPDLKNQIEVLENQVLEEESRAGSGAMVQNVVDSDTIAETAARLTGIPVNKLTESENEKLIHMERELSSEVVGQSEAIKAVSNAVRLSRSGLSNPRQPASFLFLGLSGSGKTELAKRIAAFLFNDSDAMIRVDCSELSEKYSVSKLLGTTAGYVGYEEGGFLTNQLQRRPYSVLLFDEVEKAHPDVLTVMLQMLDDGRLTSGQGKTIDCSNCIIIMTSNLGATFISSQSGSRIEESTKNLVMGAVKQHFRPEFLNRISSIVVFNKLSPKAIHRIVDIRLKEIEDRFEENDKHYKLDLTPEAKNYLARNGYSEDMGARPLNRLIQNEILNRMAMRILKGQIKDKETVRVVLKHNENPDMEEPDQLDVLPNHQSSDADMDVDNDWDEDLDDSVTDATPLD |
| *Myceliophthora*  *thermophila* | MNSKMEFTDRAKKALEDAMALAEQYAHSQLLPVHLAVALLDPLPDPSRDQQNAAPGTTSTLFRQVIERAHGDPQQFDRALKKTLVRLPSQDPPPDQVSMAPSFNTVLRKAMELQKVQKDTYIAVDHLITALAEDHTIQTALKEANIPKPKLIQDAISAIRGTKRVDSRNADAEEENENLAKFTVDMTAMAREGKLDPVIGREEEIRRVIRILSRRTKNNPVLIGEPGVGKTTVVEGLAQRIVNADVPDNLAACKLLSLDVGALVAGSKYRGEFEERMKSVLKEIEESKDMIVLFVDEIHLLMGAGSSGEGGMDAANLLKPMLARGQLHCIGATTLAEYRKYIEKDAAFERRFQQVIVKEPSIPETISILRGLKEKYEVHHGVNIADGAIVAAANLAARYLTSRRLPDSAVDLIDEAAAAVRVARESQPEIIDSLERRLRQLKIEIHALSREKDEASKARLAQAKQDAQNVEEELRPLREKYERERQRGKDIQEARLKLENLRVKAEDASRMGDHSRAADLQYYAIPEQEQIIKRLEKEKAAADAALNESGPDTGGAMVTDVVGPDQINEIVARWTGIPVTRLKTSEKERLLHMEQALSKIVVGQKEAVQSVSNAIRLQRSGLANPNQPPSFLFCGPSGTGKTLLTKALAEFLFDDPKAMIRFDMSEYQERHSLSRMIGAPPGYVGHDAGGQLTEALRRKPFSILLFDEVEKAAKEVLTVLLQLMDDGRITDGQGRIVDAKNCIVVMTSNLGAEYLSRPNGKDGKVDPTTKELVMNALRNYFLPEFLNRISSIVIFNRLTRREIRKIVDLRIAEIQKRLQDNDRNVTIRVSDAAKDKLGAAGYSPVYGARPLQRLLEKEVLNRMAILILRGSIRDGEVANVDLVDGKVTVIPNHPDSEGEDEDMMVDEDEALDEVAPDSMDEDIYD |
| *Scytalidium*  *thermophilum* | MNSKMEFTDRAKKALEDAMVLAEQYAHSQLVPVHLAISLLDPLPDPSKDQQNQAPGTTSSLFRQVVERANGDPQAFDRALKRTLVRLPSQDPPPEQVSMSPSFNKVLRQAMELQKVQKDTYIAVDHLISALSEDNTIQAALKEANIPKPKLIQEAISTIRGTRRVDSRSADTESENENLAKFTIDMTAQAREGKIDPVIGREEEIRRVIRILSRRTKNNPVLIGEPGVGKTTVVEGLAQRIVNADVPDNLANCKLLSLDVGALVAGSKYRGEFEERMKGVLKEIQESKDTIILFVDEIHLLMGAGASGEGGMDAANLLKPMLARGQLHCIGATTLAEYRKYIEKDAAFERRFQQVLVKEPTIPETISILRGLKEKYEVHHGVTIADAAIVAAANLAARYLTQRRLPDSAVDLIDEAAAAVRVARESQPEIIDSLERRLRQLKIEIHALSREKDEASKARLAQAKQDAQNVEEELRPLREKYERERQRGKAIQEAKLKLENLRVKAEEASRMGDHSRAADLQYYAIPEQEQIIKRLEREKAAADAALNESGADVGGSMITDVVGPDQINEIVARWTGIPVTRLRTSEKEKLLHMEKHLAKIVVGQKEAVQSVSNAIRLQRSGLANPNQPPSFLFCGPSGTGKTLLTKALAEFLFDDPKSMIRFDMSEYQERHSLSRMIGAPPGYVGHDAGGQLTEALRRKPFSILLFDEVEKAAKEVLTVLLQLMDDGRITDGQGRIVDAKNCIVVMTSNLGAEYLARPSGKDGKIDPTTKELVMNALRNYFLPEFLNRISSIVIFNRLTRREIRKIVDLRIAEIQKRLRDNDRNVTIIVSDAAKDKLGAAGYSPAYGARPLQRLLEKEVLNRLAILILRGNIRDGEVARVDVVDGKVTVLPNHEDEMSDEEMLVDEEDAVEEVAPDSMDEDIYD |
| *Thermoascus*  *aurantiacus* | MNGTQFTDRANKALLDSNNLAEQYAHSQILPLHLAISLLTPPQDEMEKQPAGHPSHEGAAAPLFRQVVERAHGDPQLLERNLMKMLVRQPSQDPPPERVAVSPALAKVIRQATELSKTQKDSYVAIDHLILAVVQDSQIQRALADANIPNVKLIDSAVQQIRGNKRVDSKTADAEGDNENLKKFTIDMTAMAREGKIDPVIGREEEIRRVIRILSRRTKNNPVLIGEPGVGKTTVVEGLARRIVNADVPANLAQCRLLSLDVGSLVAGSKYRGEFEERMKGVLKEIEDSKDMIVLFVDEIHLLMGAGSSGEGGMDAANLLKPMLARGQLHCIGATTLGEYRKYIEKDQAFERRFQQVLVKEPTVNETISILRGLKEKYEVHHGVNILDAAIVAAANLAARYLTARRLPDSAVDLIDEAAAAVRVTRESEPEALDNLERKLRQLQIEIHALEREQDPASKQRLEAAKQEAANVTEELRPLREKYESEKKRSKDIQDAKIKLDSLKVKRDEAERSGDTQTAADLEYYAIPETKALIERLEADRARADAEARARQGEAGETLLADAVGPDQINEIVARWTGIPVTRLKTTEKDKLLNMEKHLHRIVVGQKEAVTSVSNAIRLQRSGLSNPNSPPSFLFCGPSGTGKTLLTKALAEFLFDDPKAMIRFDMSEYQERHSLSRMIGAPPGYVGHDAGGQLTENLRRRPFSILLFDEVEKAAKEVLTVLLQLMDDGRITDGQGRIVDARNCIVVMTSNLGAEYLSRPTTKDGKIEPQTRELVMGALRDYFLPEFLNRISSIIIFNRLTKREIRKIVELRLSEVQRRLEQNDRNVKIECTEEVKDYLGDAGYSPAYGARPLSRLIEREVLNRLAVLILRGAIRDGEVARVVMREGRVEVLPNHIEPMEDEEMLDEEEALAEIEENAGGDMDLYE |
| *Arabidopsis*  *thaliana* | MNPEKFTHKTNETIATAHELAVNAGHAQFTPLHLAGALISDPTGIFPQAISSAGGENAAQSAERVINQALKKLPSQSPPPDDIPASSSLIKVIRRAQAAQKSRGDTHLAVDQLIMGLLEDSQIRDLLNEVGVATARVKSEVEKLRGKEGKKVESASGDTNFQALKTYGRDLVEQAGKLDPVIGRDEEIRRVVRILSRRTKNNPVLIGEPGVGKTAVVEGLAQRIVKGDVPNSLTDVRLISLDMGALVAGAKYRGEFEERLKSVLKEVEDAEGKVILFIDEIHLVLGAGKTEGSMDAANLFKPMLARGQLRCIGATTLEEYRKYVEKDAAFERRFQQVYVAEPSVPDTISILRGLKEKYEGHHGVRIQDRALINAAQLSARYITGRHLPDKAIDLVDEACANVRVQLDSQPEEIDNLERKRMQLEIELHALEREKDKASKARLIEVRKELDDLRDKLQPLTMKYRKEKERIDEIRRLKQKREELMFSLQEAERRYDLARAADLRYGAIQEVESAIAQLEGTSSEENVMLTENVGPEHIAEVVSRWTGIPVTRLGQNEKERLIGLADRLHKRVVGQNQAVNAVSEAILRSRAGLGRAQQPTGSFLFLGPTGVGKTELAKALAEQLFDDENLLVRIDMSEYMEQHSVSRLIGAPPGYVGHEEGGQLTEAVRRRPYCVILFDEVEKAHVAVFNTLLQVLDDGRLTDGQGRTVDFRNSVIIMTSNLGAEHLLAGLTGKVTMEVARDCVMREVRKHFRPELLNRLDEIVVFDPLSHDQLRKVARLQMKDVAVRLAERGVALAVTDAALDYILAESYDPVYGARPIRRWMEKKVVTELSKMVVREEIDENSTVYIDAGAGDLVYRVESGGLVDASTGKKSDVLIHIANGPKRSDAAQAVKKMRIEEIEDDDNEEMIED |
| *Calcarisporiella*  *thermophila* | MSSMQFTDKATETLNAAAKYAAENSHVQLHPSHVAVVMLDEENSLFRSILEKAGGDVVSIERGFKKIMVRQPSQDPPPTEMGHSPELAKLLHYAHEHMKKQRDLYIAQDHLILALADLPSMAQVLKEGGVTKKSLENAVTHVRGNRRVESKSAEEAYEALSKYCIDLTELAASGKLDPVIGRDEIISRVIRVLSRRTKNNPCLVGEPGVGKTAIAEGLANRIVKGDIPSSLQKKVYSLDIGSLLAGAKYRGEFEERLKAVLKELKEAQAIVFIDEIHTVLGAGKSEGAIDAANLLKPMLARGELRCIGATTLTEYRQYVEKDPAFERRFQLVMVEEPSVTDTISILRGLKERYETHHGVRIADAAIVAAAQLAARYITQRFMPDKAIDLIDEACANTRVQLDSQPEAIDKLERRHLQLEVEATALEKEKDAASKQRLQEVRAEMARIQEELRPLKMKYESEKGRLDEIRNLSQRLDELKAKAEDAERRYDLARAADIRYYAIPDLEKRLAQLQAEKSQADAERADGLLAEVVGPDQIMEVVSRWTGIPVSNLQRSEKEKLLHMEEYMKQHVVGQDEAIKAICDAIRLSRTGLQNRNRPLASFLFLGPTGCGKTLCVKELAAFLFNDPGAIVRIDMSEYMEKHAVSRLVGAPPGYIGHDEGGQLTEAVRRRPYTVVLFDEMEKAHKDVSNLLLQILDDGHCTDSKGRRVDFKNTIIVMTSNLGADLFELDEGDKVSQATKNAVLATARRHFANEFINRIDELIVFNRLTPSNIRKIVDVRLKEVQERLDEKQITLDVDDKAKDLLAQQGFDPVYGARPLNRLIQHALLTQLSRLLLDGGVRPGEIAKVTVDQEGEIIVIRNHGIESPAPWADEDMVEDEDMEI |
| *Plasmodium*  *falciparum* | MAPDNKQEQGKYLNRTINILNAGKNIAKSYGHNKLKPIHILSALAKSDYGSTLFKENNVNAANLKEYIDIALEQTRAGAPLDNKSKIVNSAEVKETLALAEAAANKYKSPKVDVEHLLSGLSNDELVNEIFNEVYLTDEAIKAILKRKFEKTKKDKDGKTGTLYIEQFGSNMNEKVRNGKLQGIYGRDEEIRAIIESLLRYNKNSPVLVGNPGTGKTTIVEGLVYRIEKGDVPKELQGYTVISLNFRKFTSGTSYRGEFETRMKNIIKELKNKKNKIILFVDEIHLLLGAGKAEGGTDAANLLKPVLSKGEIKLIGATTIAEYRKFIESCSAFERRFEKILVEPPSVDMTVKILRSLKSKYENFYGINITDKALVAAAKISDRFIKDRYLPDKAIDLLNKACSFLQVQLSGKPRIIDVTERDIERLSYEISTLEKDVDKVSKKKYNKLIKEFEEKKEQLKKYYEEYVITGERLKRKKEIEKKLNDLKELTQNYVYSNKEPPIELQNSLKEAQQKYLELYKETVAYVEAKTHNAMNVDAVYQEHVSYIYLRDSGMPLGSLSFESSKGALKLYNSLSKSIIGNEDIIKSLSDAVVKAATGMKDPEKPIGTFLFLGPTGVGKTELAKTLAIELFNSKDNLIRVNMSEFTEAHSVSKITGSPPGYVGFSDSGQLTEAVREKPHSVVLFDELEKAHADVFKVLLQILGDGYINDNHRRNIDFSNTIIIMTSNLGAELFKKKLFFDADNSGTPEYKRVMEDVRLSLIKKCKKVFKPEFVNRIDKIGVFEPLNKKNLHKIVALRFKKLEKRLEEKNIQVSVSEKAIDYIIDQSYDPELGARPTLIFIESVIMTKFAIMYLKKELVDDMDVFVDYNSKAKNLVINLSKT |
