# Supplementary material for: Therapeutic genetic variation revealed in diverse Hsp104 homologs
Source: eLife. 2020 Dec 15;9:e57457. doi: 10.7554/eLife.57457 (PMC7785292; doi:10.7554/eLife.57457)
Supplement: Supplementary file 2. — The pairwise sequence identities between Hsp104 homologs were calculated using UniProt Align tool. [file elife-57457-supp2.docx]

**Supplementary File 2. Pairwise sequence identity between Hsp104 homologs.**

|  |  | Total aa | Identical aa | % Identity | Similar aa |
| --- | --- | --- | --- | --- | --- |
|  | *S.cerevisiae* | 908 |  |  |  |
| *S.cerevisiae* | *P.falciparum* | 881 | 319 | 34 | 316 |
| *S.cerevisiae* | *L.thermotolerans* | 907 | 761 | 84 | 105 |
| *S.cerevisiae* | *Ch.thermophilum* | 926 | 467 | 50 | 265 |
| *S.cerevisiae* | *S.thermophilum* | 924 | 463 | 49 | 265 |
| *S.cerevisiae* | *M.thermophila* | 925 | 459 | 49 | 274 |
| *S.cerevisiae* | *T.terrestris* | 923 | 463 | 49 | 262 |
| *S.cerevisiae* | *T.lanuginosus* | 922 | 454 | 48 | 294 |
| *S.cerevisiae* | *T.aurantiacus* | 926 | 458 | 49 | 291 |
| *S.cerevisiae* | *C.thermophila* | 882 | 418 | 46 | 292 |
| *S.cerevisiae* | *C.reinhardtii* | 925 | 383 | 40 | 298 |
| *S.cerevisiae* | *P.euphratica* | 914 | 391 | 42 | 298 |
| *S.cerevisiae* | *A.thaliana* | 911 | 394 | 42 | 294 |
| *S.cerevisiae* | *M.brevicollis* | 889 | 387 | 42 | 303 |
| *S.cerevisiae* | *S.rosetta* | 892 | 381 | 41 | 316 |
| *S.cerevisiae* | *G.sulphuraria* | 922 | 386 | 41 | 332 |
| *S.cerevisiae* | *D.discoideum* | 886 | 401 | 43 | 299 |
| *P.falciparum* | *L.thermotolerans* |  | 302 | 32 | 337 |
| *P.falciparum* | *Ch.thermophilum* |  | 317 | 33 | 331 |
| *P.falciparum* | *S.thermophilum* |  | 316 | 34 | 328 |
| *P.falciparum* | *M.thermophila* |  | 322 | 34 | 321 |
| *P.falciparum* | *T.terrestris* |  | 319 | 34 | 323 |
| *P.falciparum* | *T.lanuginosus* |  | 304 | 32 | 326 |
| *P.falciparum* | *T.aurantiacus* |  | 307 | 32 | 324 |
| *P.falciparum* | *C.thermophila* |  | 306 | 33 | 330 |
| *P.falciparum* | *C.reinhardtii* |  | 318 | 34 | 321 |
| *P.falciparum* | *P.euphratica* |  | 326 | 35 | 334 |
| *P.falciparum* | *A.thaliana* |  | 329 | 35 | 327 |
| *P.falciparum* | *M.brevicollis* |  | 321 | 35 | 332 |
| *P.falciparum* | *S.rosetta* |  | 316 | 35 | 337 |
| *P.falciparum* | *G.sulphuraria* |  | 341 | 36 | 324 |
| *P.falciparum* | *D.discoideum* |  | 328 | 36 | 343 |
| *L.thermotolerans* | *Ch.thermophilum* |  | 469 | 50 | 269 |
| *L.thermotolerans* | *S.thermophilum* |  | 459 | 49 | 279 |
| *L.thermotolerans* | *M.thermophila* |  | 465 | 50 | 273 |
| *L.thermotolerans* | *T.terrestris* |  | 459 | 49 | 272 |
| *L.thermotolerans* | *T.lanuginosus* |  | 448 | 48 | 300 |
| *L.thermotolerans* | *T.aurantiacus* |  | 450 | 48 | 298 |
| *L.thermotolerans* | *C.thermophila* |  | 411 | 45 | 298 |
| *L.thermotolerans* | *C.reinhardtii* |  | 367 | 39 | 315 |
| *L.thermotolerans* | *P.euphratica* |  | 390 | 42 | 310 |
| *L.thermotolerans* | *A.thaliana* |  | 391 | 42 | 302 |
| *L.thermotolerans* | *M.brevicollis* |  | 387 | 42 | 309 |
| *L.thermotolerans* | *S.rosetta* |  | 381 | 41 | 312 |
| *L.thermotolerans* | *G.sulphuraria* |  | 405 | 43 | 314 |
| *L.thermotolerans* | *D.discoideum* |  | 410 | 44 | 295 |
| *Ch.thermophilum* | *S.thermophilum* |  | 831 | 90 | 61 |
| *Ch.thermophilum* | *M.thermophila* |  | 841 | 91 | 54 |
| *Ch.thermophilum* | *T.terrestris* |  | 834 | 90 | 51 |
| *Ch.thermophilum* | *T.lanuginosus* |  | 697 | 75 | 147 |
| *Ch.thermophilum* | *T.aurantiacus* |  | 692 | 74 | 142 |
| *Ch.thermophilum* | *C.thermophila* |  | 486 | 52 | 258 |
| *Ch.thermophilum* | *C.reinhardtii* |  | 420 | 43 | 283 |
| *Ch.thermophilum* | *P.euphratica* |  | 452 | 48 | 271 |
| *Ch.thermophilum* | *A.thaliana* |  | 449 | 47 | 278 |
| *Ch.thermophilum* | *M.brevicollis* |  | 438 | 47 | 278 |
| *Ch.thermophilum* | *S.rosetta* |  | 430 | 46 | 280 |
| *Ch.thermophilum* | *G.sulphuraria* |  | 460 | 48 | 276 |
| *Ch.thermophilum* | *D.discoideum* |  | 446 | 47 | 278 |
| *S.thermophilum* | *M.thermophila* |  | 850 | 92 | 63 |
| *S.thermophilum* | *T.terrestris* |  | 840 | 91 | 60 |
| *S.thermophilum* | *T.lanuginosus* |  | 697 | 75 | 147 |
| *S.thermophilum* | *T.aurantiacus* |  | 703 | 76 | 132 |
| *S.thermophilum* | *C.thermophila* |  | 483 | 52 | 252 |
| *S.thermophilum* | *C.reinhardtii* |  | 424 | 44 | 281 |
| *S.thermophilum* | *P.euphratica* |  | 445 | 47 | 288 |
| *S.thermophilum* | *A.thaliana* |  | 458 | 48 | 273 |
| *S.thermophilum* | *M.brevicollis* |  | 440 | 48 | 276 |
| *S.thermophilum* | *S.rosetta* |  | 431 | 46 | 278 |
| *S.thermophilum* | *G.sulphuraria* |  | 463 | 49 | 278 |
| *S.thermophilum* | *D.discoideum* |  | 447 | 48 | 284 |
| *M.thermophila* | *T.terrestris* |  | 848 | 91 | 58 |
| *M.thermophila* | *T.lanuginosus* |  | 701 | 76 | 145 |
| *M.thermophila* | *T.aurantiacus* |  | 693 | 75 | 146 |
| *M.thermophila* | *C.thermophila* |  | 485 | 52 | 254 |
| *M.thermophila* | *C.reinhardtii* |  | 423 | 44 | 286 |
| *M.thermophila* | *P.euphratica* |  | 445 | 47 | 271 |
| *M.thermophila* | *A.thaliana* |  | 458 | 48 | 268 |
| *M.thermophila* | *M.brevicollis* |  | 439 | 47 | 280 |
| *M.thermophila* | *S.rosetta* |  | 432 | 46 | 275 |
| *M.thermophila* | *G.sulphuraria* |  | 457 | 48 | 282 |
| *M.thermophila* | *D.discoideum* |  | 451 | 48 | 281 |
| *T.terrestris* | *T.lanuginosus* |  | 698 | 75 | 143 |
| *T.terrestris* | *T.aurantiacus* |  | 695 | 75 | 141 |
| *T.terrestris* | *C.thermophila* |  | 483 | 52 | 251 |
| *T.terrestris* | *C.reinhardtii* |  | 427 | 45 | 280 |
| *T.terrestris* | *P.euphratica* |  | 447 | 47 | 277 |
| *T.terrestris* | *A.thaliana* |  | 454 | 48 | 274 |
| *T.terrestris* | *M.brevicollis* |  | 443 | 48 | 271 |
| *T.terrestris* | *S.rosetta* |  | 427 | 46 | 280 |
| *T.terrestris* | *G.sulphuraria* |  | 458 | 48 | 284 |
| *T.terrestris* | *D.discoideum* |  | 444 | 47 | 279 |
| *T.lanuginosus* | *T.aurantiacus* |  | 794 | 85 | 101 |
| *T.lanuginosus* | *C.thermophila* |  | 482 | 52 | 256 |
| *T.lanuginosus* | *C.reinhardtii* |  | 418 | 43 | 276 |
| *T.lanuginosus* | *P.euphratica* |  | 437 | 46 | 275 |
| *T.lanuginosus* | *A.thaliana* |  | 447 | 47 | 265 |
| *T.lanuginosus* | *M.brevicollis* |  | 437 | 47 | 277 |
| *T.lanuginosus* | *S.rosetta* |  | 420 | 25 | 290 |
| *T.lanuginosus* | *G.sulphuraria* |  | 460 | 49 | 281 |
| *T.lanuginosus* | *D.discoideum* |  | 444 | 48 | 292 |
| *T.aurantiacus* | *C.thermophila* |  | 475 | 51 | 261 |
| *T.aurantiacus* | *C.reinhardtii* |  | 424 | 44 | 271 |
| *T.aurantiacus* | *P.euphratica* |  | 444 | 47 | 270 |
| *T.aurantiacus* | *A.thaliana* |  | 446 | 47 | 267 |
| *T.aurantiacus* | *M.brevicollis* |  | 419 | 45 | 293 |
| *T.aurantiacus* | *S.rosetta* |  | 417 | 45 | 293 |
| *T.aurantiacus* | *G.sulphuraria* |  | 456 | 48 | 281 |
| *T.aurantiacus* | *D.discoideum* |  | 439 | 47 | 300 |
| *C.thermophila* | *C.reinhardtii* |  | 454 | 49 | 257 |
| *C.thermophila* | *P.euphratica* |  | 463 | 50 | 257 |
| *C.thermophila* | *A.thaliana* |  | 464 | 50 | 253 |
| *C.thermophila* | *M.brevicollis* |  | 468 | 52 | 258 |
| *C.thermophila* | *S.rosetta* |  | 467 | 52 | 259 |
| *C.thermophila* | *G.sulphuraria* |  | 479 | 52 | 264 |
| *C.thermophila* | *D.discoideum* |  | 494 | 55 | 263 |
| *C.reinhardtii* | *P.euphratica* |  | 526 | 56 | 236 |
| *C.reinhardtii* | *A.thaliana* |  | 523 | 56 | 242 |
| *C.reinhardtii* | *M.brevicollis* |  | 487 | 52 | 248 |
| *C.reinhardtii* | *S.rosetta* |  | 480 | 51 | 245 |
| *C.reinhardtii* | *G.sulphuraria* |  | 503 | 53 | 236 |
| *C.reinhardtii* | *D.discoideum* |  | 491 | 53 | 248 |
| *P.euphratica* | *A.thaliana* |  | 791 | 86 | 97 |
| *P.euphratica* | *M.brevicollis* |  | 500 | 54 | 252 |
| *P.euphratica* | *S.rosetta* |  | 487 | 52 | 261 |
| *P.euphratica* | *G.sulphuraria* |  | 526 | 55 | 242 |
| *P.euphratica* | *D.discoideum* |  | 525 | 57 | 235 |
| *A.thaliana* | *M.brevicollis* |  | 507 | 54 | 236 |
| *A.thaliana* | *S.rosetta* |  | 500 | 54 | 255 |
| *A.thaliana* | *G.sulphuraria* |  | 527 | 56 | 242 |
| *A.thaliana* | *D.discoideum* |  | 527 | 57 | 231 |
| *M.brevicollis* | *S.rosetta* |  | 634 | 71 | 176 |
| *M.brevicollis* | *G.sulphuraria* |  | 528 | 57 | 225 |
| *M.brevicollis* | *D.discoideum* |  | 512 | 57 | 255 |
| *S.rosetta* | *G.sulphuraria* |  | 517 | 56 | 240 |
| *S.rosetta* | *D.discoideum* |  | 499 | 56 | 282 |
| *G.sulphuraria* | *D.discoideum* |  | 538 | 58 | 240 |
