# Supplementary material for: Therapeutic genetic variation revealed in diverse Hsp104 homologs
Source: eLife. 2020 Dec 15;9:e57457. doi: 10.7554/eLife.57457 (PMC7785292; doi:10.7554/eLife.57457)
Supplement: Supplementary file 3. — The primary sequence of each Hsp104 chimera is provided. [file elife-57457-supp3.docx]

**Supplementary File 3. Amino acid sequences of Hsp104 chimeras.**

| **Chimera Name** | **Sequence** |
| --- | --- |
| GSSS | MNLDNLTDKAQEAIQASHELALENGHSQLTPLHLAAALFTEDHGLASSVATKAKADPVNVRRELQKAVIRLPSQDPPPTTVPPSQSFLKVIRDAQSLRKKQGDTHLAVDHLLIALCDDKDVIACFSSADLTKHALEEAVKSVRGNTRIDSRGADTNTPLEYLSKYAIDMTEQARQGKLDPVIGREEEIRSTIRVLARRIKSNPCLIGEPGIGKTAIIEGVAQRIIDDDVPTILQGAKLFSLDLAALTAGAKYKGDFEERFKGVLKEIEESKTLIVLFIDEIHMLMGNGKDDAANILKPALSRGQLKVIGATTNNEYRSIVEKDGAFERRFQKIEVAEPSVRQTVAILRGLQPKYEIHHGVRILDSALVTAAQLAKRYLPYRRLPDSALDLVDISCAGVAVARDSKPEELDSKERQLQLIQVEIKALERDEDADSTTKDRLKLARQKEASLQEELEPLRQRYNEEKHGHEELTQAKKKLDELENKALDAERRYDTATAADLRYFAIPDIKKQIEKLEDQVAEEERRAGANSMIQNVVDSDTISETAARLTGIPVKKLSESENEKLIHMERDLSSEVVGQMDAIKAVSNAVRLSRSGLANPRQPASFLFLGLSGSGKTELAKKVAGFLFNDEDMMIRVDCSELSEKYAVSKLLGTTAGYVGYDEGGFLTNQLQYKPYSVLLFDEVEKAHPDVLTVMLQMLDDGRITSGQGKTIDCSNCIVIMTSNLGAEFINSQQGSKIQESTKNLVMGAVRQHFRPEFLNRISSIVIFNKLSRKAIHKIVDIRLKEIEERFEQNDKHYKLNLTQEAKDFLAKYGYSDDMGARPLNRLIQNEILNKLALRILKNEIKDKETVNVVLKKGKSRDENVPEEAEECLEVLPNHEATIGADTLGDDDNEDSMEIDDDLD |
| GGSS | MNLDNLTDKAQEAIQASHELALENGHSQLTPLHLAAALFTEDHGLASSVATKAKADPVNVRRELQKAVIRLPSQDPPPTTVPPSQSFLKVIRDAQSLRKKQGDTHLAVDHLLIALCDDKDVIACFSSADLTKHALEEAVKSVRGNRKVDSKAADSTYDALNQYAQDFVALAEEGKLDPVIGRDDEIRRVIRVLCRRRKNNPVLIGDPGVGKTAIVEGLAQRIVRGDVPENLNCRLYALDMGALVAGAKYRGEFEERLKAVLREVKEGEGKIILFIDELHLVLGAGKSDGAMDAANLLKPMLARGELRCIGATTLEEYRKYVEKDAAFERRFQQVFVSEPSVPDTVSILRGLKERYEVHHGVRILDSALVAAAKLSARYITNRFLPDKAIDLVDEACANVRMQLDSKPEELDSKERQLQLIQVEIKALERDEDADSTTKDRLKLARQKEASLQEELEPLRQRYNEEKHGHEELTQAKKKLDELENKALDAERRYDTATAADLRYFAIPDIKKQIEKLEDQVAEEERRAGANSMIQNVVDSDTISETAARLTGIPVKKLSESENEKLIHMERDLSSEVVGQMDAIKAVSNAVRLSRSGLANPRQPASFLFLGLSGSGKTELAKKVAGFLFNDEDMMIRVDCSELSEKYAVSKLLGTTAGYVGYDEGGFLTNQLQYKPYSVLLFDEVEKAHPDVLTVMLQMLDDGRITSGQGKTIDCSNCIVIMTSNLGAEFINSQQGSKIQESTKNLVMGAVRQHFRPEFLNRISSIVIFNKLSRKAIHKIVDIRLKEIEERFEQNDKHYKLNLTQEAKDFLAKYGYSDDMGARPLNRLIQNEILNKLALRILKNEIKDKETVNVVLKKGKSRDENVPEEAEECLEVLPNHEATIGADTLGDDDNEDSMEIDDDLD |
| GGGS | MNLDNLTDKAQEAIQASHELALENGHSQLTPLHLAAALFTEDHGLASSVATKAKADPVNVRRELQKAVIRLPSQDPPPTTVPPSQSFLKVIRDAQSLRKKQGDTHLAVDHLLIALCDDKDVIACFSSADLTKHALEEAVKSVRGNRKVDSKAADSTYDALNQYAQDFVALAEEGKLDPVIGRDDEIRRVIRVLCRRRKNNPVLIGDPGVGKTAIVEGLAQRIVRGDVPENLNCRLYALDMGALVAGAKYRGEFEERLKAVLREVKEGEGKIILFIDELHLVLGAGKSDGAMDAANLLKPMLARGELRCIGATTLEEYRKYVEKDAAFERRFQQVFVSEPSVPDTVSILRGLKERYEVHHGVRILDSALVAAAKLSARYITNRFLPDKAIDLVDEACANVRMQLDSQPEVIDTLEHRKLQLEIEIAALEKEKDEASRARLAAVKEELDNVNEKLRPLKARFESERGKMNELKDMMTKLDALKIKLADAERRRDTVQAADLRYYAIPEIEERIRSLKNEIDKEKETDAMETDEESGKLLSDVVGYEQIADVVSRLTGIPVKKLSESENEKLIHMERDLSSEVVGQMDAIKAVSNAVRLSRSGLANPRQPASFLFLGLSGSGKTELAKKVAGFLFNDEDMMIRVDCSELSEKYAVSKLLGTTAGYVGYDEGGFLTNQLQYKPYSVLLFDEVEKAHPDVLTVMLQMLDDGRITSGQGKTIDCSNCIVIMTSNLGAEFINSQQGSKIQESTKNLVMGAVRQHFRPEFLNRISSIVIFNKLSRKAIHKIVDIRLKEIEERFEQNDKHYKLNLTQEAKDFLAKYGYSDDMGARPLNRLIQNEILNKLALRILKNEIKDKETVNVVLKKGKSRDENVPEEAEECLEVLPNHEATIGADTLGDDDNEDSMEIDDDLD |
| SSGS | MNDQTQFTERALTILTLAQKLASDHQHPQLQPIHILAAFIETPEDGSVPYLQNLIEKGRYDYDLFKKVVNRNLVRIPQQQPAPAEITPSYALGKVLQDAAKIQKQQKDSFIAQDHILFALFNDSSIQQIFKEAQVDIEAIKQQALELRGNTRIDSRGADTNTPLEYLSKYAIDMTEQARQGKLDPVIGREEEIRSTIRVLARRIKSNPCLIGEPGIGKTAIIEGVAQRIIDDDVPTILQGAKLFSLDLAALTAGAKYKGDFEERFKGVLKEIEESKTLIVLFIDEIHMLMGNGKDDAANILKPALSRGQLKVIGATTNNEYRSIVEKDGAFERRFQKIEVAEPSVRQTVAILRGLQPKYEIHHGVRILDSALVTAAQLAKRYLPYRRLPDSALDLVDISCAGVAVARDSQPEVIDTLEHRKLQLEIEIAALEKEKDEASRARLAAVKEELDNVNEKLRPLKARFESERGKMNELKDMMTKLDALKIKLADAERRRDTVQAADLRYYAIPEIEERIRSLKNEIDKEKETDAMETDEESGKLLSDVVGYEQIADVVSRLTGIPVKKLSESENEKLIHMERDLSSEVVGQMDAIKAVSNAVRLSRSGLANPRQPASFLFLGLSGSGKTELAKKVAGFLFNDEDMMIRVDCSELSEKYAVSKLLGTTAGYVGYDEGGFLTNQLQYKPYSVLLFDEVEKAHPDVLTVMLQMLDDGRITSGQGKTIDCSNCIVIMTSNLGAEFINSQQGSKIQESTKNLVMGAVRQHFRPEFLNRISSIVIFNKLSRKAIHKIVDIRLKEIEERFEQNDKHYKLNLTQEAKDFLAKYGYSDDMGARPLNRLIQNEILNKLALRILKNEIKDKETVNVVLKKGKSRDENVPEEAEECLEVLPNHEATIGADTLGDDDNEDSMEIDDDLD |
| GSGS | MNLDNLTDKAQEAIQASHELALENGHSQLTPLHLAAALFTEDHGLASSVATKAKADPVNVRRELQKAVIRLPSQDPPPTTVPPSQSFLKVIRDAQSLRKKQGDTHLAVDHLLIALCDDKDVIACFSSADLTKHALEEAVKSVRGNTRIDSRGADTNTPLEYLSKYAIDMTEQARQGKLDPVIGREEEIRSTIRVLARRIKSNPCLIGEPGIGKTAIIEGVAQRIIDDDVPTILQGAKLFSLDLAALTAGAKYKGDFEERFKGVLKEIEESKTLIVLFIDEIHMLMGNGKDDAANILKPALSRGQLKVIGATTNNEYRSIVEKDGAFERRFQKIEVAEPSVRQTVAILRGLQPKYEIHHGVRILDSALVTAAQLAKRYLPYRRLPDSALDLVDISCAGVAVARDSQPEVIDTLEHRKLQLEIEIAALEKEKDEASRARLAAVKEELDNVNEKLRPLKARFESERGKMNELKDMMTKLDALKIKLADAERRRDTVQAADLRYYAIPEIEERIRSLKNEIDKEKETDAMETDEESGKLLSDVVGYEQIADVVSRLTGIPVKKLSESENEKLIHMERDLSSEVVGQMDAIKAVSNAVRLSRSGLANPRQPASFLFLGLSGSGKTELAKKVAGFLFNDEDMMIRVDCSELSEKYAVSKLLGTTAGYVGYDEGGFLTNQLQYKPYSVLLFDEVEKAHPDVLTVMLQMLDDGRITSGQGKTIDCSNCIVIMTSNLGAEFINSQQGSKIQESTKNLVMGAVRQHFRPEFLNRISSIVIFNKLSRKAIHKIVDIRLKEIEERFEQNDKHYKLNLTQEAKDFLAKYGYSDDMGARPLNRLIQNEILNKLALRILKNEIKDKETVNVVLKKGKSRDENVPEEAEECLEVLPNHEATIGADTLGDDDNEDSMEIDDDLD |
| MSSS | MAINPNEFTDKVNKTLFEAQNFAIQEGHSQVEPAHVAVILFEDPEGMAKRVVQRAGAALQPVQAALRSLLQRMPRQEPAPLEASLSSDTRRLLQSAAKLQKKNNEAHLAVDHLLGALVQDKQILAKLAESGLAKNHFEETLKRVKRGNTRIDSRGADTNTPLEYLSKYAIDMTEQARQGKLDPVIGREEEIRSTIRVLARRIKSNPCLIGEPGIGKTAIIEGVAQRIIDDDVPTILQGAKLFSLDLAALTAGAKYKGDFEERFKGVLKEIEESKTLIVLFIDEIHMLMGNGKDDAANILKPALSRGQLKVIGATTNNEYRSIVEKDGAFERRFQKIEVAEPSVRQTVAILRGLQPKYEIHHGVRILDSALVTAAQLAKRYLPYRRLPDSALDLVDISCAGVAVARDSKPEELDSKERQLQLIQVEIKALERDEDADSTTKDRLKLARQKEASLQEELEPLRQRYNEEKHGHEELTQAKKKLDELENKALDAERRYDTATAADLRYFAIPDIKKQIEKLEDQVAEEERRAGANSMIQNVVDSDTISETAARLTGIPVKKLSESENEKLIHMERDLSSEVVGQMDAIKAVSNAVRLSRSGLANPRQPASFLFLGLSGSGKTELAKKVAGFLFNDEDMMIRVDCSELSEKYAVSKLLGTTAGYVGYDEGGFLTNQLQYKPYSVLLFDEVEKAHPDVLTVMLQMLDDGRITSGQGKTIDCSNCIVIMTSNLGAEFINSQQGSKIQESTKNLVMGAVRQHFRPEFLNRISSIVIFNKLSRKAIHKIVDIRLKEIEERFEQNDKHYKLNLTQEAKDFLAKYGYSDDMGARPLNRLIQNEILNKLALRILKNEIKDKETVNVVLKKGKSRDENVPEEAEECLEVLPNHEATIGADTLGDDDNEDSMEIDDDLD |
| MMSS | MAINPNEFTDKVNKTLFEAQNFAIQEGHSQVEPAHVAVILFEDPEGMAKRVVQRAGAALQPVQAALRSLLQRMPRQEPAPLEASLSSDTRRLLQSAAKLQKKNNEAHLAVDHLLGALVQDKQILAKLAESGLAKNHFEETLKRVKGTTTADSKSAEENYDALSKYGVDLVQQAADGKLDPVLGRDEEIRRVIQILARRIKSNPCLVGPPGVGKSAIVEGLAQRIMLGDVPETLKGKLISLDMGALIAGAKYRGEFEERLKAVLEEIKQSEGRIILFVDEVHNVLGAGKTEGSMDAANLLKPLLARGELRMIGATTEDEYRKYVEKDSAFERRFQVVQVREPSVPDTVSILRGLKERYEAHHGVRIADAALVAAAQLSHRYIQGRFLPDKAIDLIDEACANARVQLDSKPEELDSKERQLQLIQVEIKALERDEDADSTTKDRLKLARQKEASLQEELEPLRQRYNEEKHGHEELTQAKKKLDELENKALDAERRYDTATAADLRYFAIPDIKKQIEKLEDQVAEEERRAGANSMIQNVVDSDTISETAARLTGIPVKKLSESENEKLIHMERDLSSEVVGQMDAIKAVSNAVRLSRSGLANPRQPASFLFLGLSGSGKTELAKKVAGFLFNDEDMMIRVDCSELSEKYAVSKLLGTTAGYVGYDEGGFLTNQLQYKPYSVLLFDEVEKAHPDVLTVMLQMLDDGRITSGQGKTIDCSNCIVIMTSNLGAEFINSQQGSKIQESTKNLVMGAVRQHFRPEFLNRISSIVIFNKLSRKAIHKIVDIRLKEIEERFEQNDKHYKLNLTQEAKDFLAKYGYSDDMGARPLNRLIQNEILNKLALRILKNEIKDKETVNVVLKKGKSRDENVPEEAEECLEVLPNHEATIGADTLGDDDNEDSMEIDDDLD |
| MMMS | MAINPNEFTDKVNKTLFEAQNFAIQEGHSQVEPAHVAVILFEDPEGMAKRVVQRAGAALQPVQAALRSLLQRMPRQEPAPLEASLSSDTRRLLQSAAKLQKKNNEAHLAVDHLLGALVQDKQILAKLAESGLAKNHFEETLKRVKGTTTADSKSAEENYDALSKYGVDLVQQAADGKLDPVLGRDEEIRRVIQILARRIKSNPCLVGPPGVGKSAIVEGLAQRIMLGDVPETLKGKLISLDMGALIAGAKYRGEFEERLKAVLEEIKQSEGRIILFVDEVHNVLGAGKTEGSMDAANLLKPLLARGELRMIGATTEDEYRKYVEKDSAFERRFQVVQVREPSVPDTVSILRGLKERYEAHHGVRIADAALVAAAQLSHRYIQGRFLPDKAIDLIDEACANARVQLDSRPEEIDQLERRRLQLQVEATALEKEKDQASKLRLKDVRKELANIEEQLQPLLMKFEMERGRVDELRDLQEKLDSLRSKAQRAERQGDLATAADLKYYAIPDCERRIQQLTLEDEERSAQRSGMDEQEDAPMLSEEVGPEQITDIIARLTGIPVKKLSESENEKLIHMERDLSSEVVGQMDAIKAVSNAVRLSRSGLANPRQPASFLFLGLSGSGKTELAKKVAGFLFNDEDMMIRVDCSELSEKYAVSKLLGTTAGYVGYDEGGFLTNQLQYKPYSVLLFDEVEKAHPDVLTVMLQMLDDGRITSGQGKTIDCSNCIVIMTSNLGAEFINSQQGSKIQESTKNLVMGAVRQHFRPEFLNRISSIVIFNKLSRKAIHKIVDIRLKEIEERFEQNDKHYKLNLTQEAKDFLAKYGYSDDMGARPLNRLIQNEILNKLALRILKNEIKDKETVNVVLKKGKSRDENVPEEAEECLEVLPNHEATIGADTLGDDDNEDSMEIDDDLD |
| SSMS | MNDQTQFTERALTILTLAQKLASDHQHPQLQPIHILAAFIETPEDGSVPYLQNLIEKGRYDYDLFKKVVNRNLVRIPQQQPAPAEITPSYALGKVLQDAAKIQKQQKDSFIAQDHILFALFNDSSIQQIFKEAQVDIEAIKQQALELRGNTRIDSRGADTNTPLEYLSKYAIDMTEQARQGKLDPVIGREEEIRSTIRVLARRIKSNPCLIGEPGIGKTAIIEGVAQRIIDDDVPTILQGAKLFSLDLAALTAGAKYKGDFEERFKGVLKEIEESKTLIVLFIDEIHMLMGNGKDDAANILKPALSRGQLKVIGATTNNEYRSIVEKDGAFERRFQKIEVAEPSVRQTVAILRGLQPKYEIHHGVRILDSALVTAAQLAKRYLPYRRLPDSALDLVDISCAGVAVARDSKPEEIDQLERRRLQLQVEATALEKEKDQASKLRLKDVRKELANIEEQLQPLLMKFEMERGRVDELRDLQEKLDSLRSKAQRAERQGDLATAADLKYYAIPDCERRIQQLTLEDEERSAQRSGMDEQEDAPMLSEEVGPEQITDIIARLTGIPVKKLSESENEKLIHMERDLSSEVVGQMDAIKAVSNAVRLSRSGLANPRQPASFLFLGLSGSGKTELAKKVAGFLFNDEDMMIRVDCSELSEKYAVSKLLGTTAGYVGYDEGGFLTNQLQYKPYSVLLFDEVEKAHPDVLTVMLQMLDDGRITSGQGKTIDCSNCIVIMTSNLGAEFINSQQGSKIQESTKNLVMGAVRQHFRPEFLNRISSIVIFNKLSRKAIHKIVDIRLKEIEERFEQNDKHYKLNLTQEAKDFLAKYGYSDDMGARPLNRLIQNEILNKLALRILKNEIKDKETVNVVLKKGKSRDENVPEEAEECLEVLPNHEATIGADTLGDDDNEDSMEIDDDLD |
| MSMS | MAINPNEFTDKVNKTLFEAQNFAIQEGHSQVEPAHVAVILFEDPEGMAKRVVQRAGAALQPVQAALRSLLQRMPRQEPAPLEASLSSDTRRLLQSAAKLQKKNNEAHLAVDHLLGALVQDKQILAKLAESGLAKNHFEETLKRVKRGNTRIDSRGADTNTPLEYLSKYAIDMTEQARQGKLDPVIGREEEIRSTIRVLARRIKSNPCLIGEPGIGKTAIIEGVAQRIIDDDVPTILQGAKLFSLDLAALTAGAKYKGDFEERFKGVLKEIEESKTLIVLFIDEIHMLMGNGKDDAANILKPALSRGQLKVIGATTNNEYRSIVEKDGAFERRFQKIEVAEPSVRQTVAILRGLQPKYEIHHGVRILDSALVTAAQLAKRYLPYRRLPDSALDLVDISCAGVAVARDSKPEEIDQLERRRLQLQVEATALEKEKDQASKLRLKDVRKELANIEEQLQPLLMKFEMERGRVDELRDLQEKLDSLRSKAQRAERQGDLATAADLKYYAIPDCERRIQQLTLEDEERSAQRSGMDEQEDAPMLSEEVGPEQITDIIARLTGIPVKKLSESENEKLIHMERDLSSEVVGQMDAIKAVSNAVRLSRSGLANPRQPASFLFLGLSGSGKTELAKKVAGFLFNDEDMMIRVDCSELSEKYAVSKLLGTTAGYVGYDEGGFLTNQLQYKPYSVLLFDEVEKAHPDVLTVMLQMLDDGRITSGQGKTIDCSNCIVIMTSNLGAEFINSQQGSKIQESTKNLVMGAVRQHFRPEFLNRISSIVIFNKLSRKAIHKIVDIRLKEIEERFEQNDKHYKLNLTQEAKDFLAKYGYSDDMGARPLNRLIQNEILNKLALRILKNEIKDKETVNVVLKKGKSRDENVPEEAEECLEVLPNHEATIGADTLGDDDNEDSMEIDDDLD |
| CSSS | MSFDTKKATEKVNNVLGEAINLAKEDKHAALTPTHLAVVLFEEPHGLAKVAATKVAGEEVWRSAIRVLRKRLTKLPKVDPAPESVSPGRELSKVLTAAAKLQKDRGDAFLGTDTLLTAVINAAEVSEALGEAGISKAQLETALSEVRQARGNTRIDSRGADTNTPLEYLSKYAIDMTEQARQGKLDPVIGREEEIRSTIRVLARRIKSNPCLIGEPGIGKTAIIEGVAQRIIDDDVPTILQGAKLFSLDLAALTAGAKYKGDFEERFKGVLKEIEESKTLIVLFIDEIHMLMGNGKDDAANILKPALSRGQLKVIGATTNNEYRSIVEKDGAFERRFQKIEVAEPSVRQTVAILRGLQPKYEIHHGVRILDSALVTAAQLAKRYLPYRRLPDSALDLVDISCAGVAVARDSKPEELDSKERQLQLIQVEIKALERDEDADSTTKDRLKLARQKEASLQEELEPLRQRYNEEKHGHEELTQAKKKLDELENKALDAERRYDTATAADLRYFAIPDIKKQIEKLEDQVAEEERRAGANSMIQNVVDSDTISETAARLTGIPVKKLSESENEKLIHMERDLSSEVVGQMDAIKAVSNAVRLSRSGLANPRQPASFLFLGLSGSGKTELAKKVAGFLFNDEDMMIRVDCSELSEKYAVSKLLGTTAGYVGYDEGGFLTNQLQYKPYSVLLFDEVEKAHPDVLTVMLQMLDDGRITSGQGKTIDCSNCIVIMTSNLGAEFINSQQGSKIQESTKNLVMGAVRQHFRPEFLNRISSIVIFNKLSRKAIHKIVDIRLKEIEERFEQNDKHYKLNLTQEAKDFLAKYGYSDDMGARPLNRLIQNEILNKLALRILKNEIKDKETVNVVLKKGKSRDENVPEEAEECLEVLPNHEATIGADTLGDDDNEDSMEIDDDLD |
| CCSS | MSFDTKKATEKVNNVLGEAINLAKEDKHAALTPTHLAVVLFEEPHGLAKVAATKVAGEEVWRSAIRVLRKRLTKLPKVDPAPESVSPGRELSKVLTAAAKLQKDRGDAFLGTDTLLTAVINAAEVSEALGEAGISKAQLETALSEVRQAAGGGPINSETADANFDALAKYGTDLTANAARADPVIGRDDEIRRVVRVLCRRTKNNPVLIGEPGVGKTAIVEGLAQRIVKNDVPETLQGVRLISLDMGSLVAGAKYRGEFEERLKAVLNEVAQQQGKVVLFIDELHLVLGAGKSGDGAMDAANLLKPMLARGELRCIGATTLGEYREHIEKDAAFERRFQQVLVKEPSVPDTIAILRGIKDRYETHHGVHITDRALVVAAELSDRYITTRFLPDKAIDLVDEACANMRVQLDSKPEELDSKERQLQLIQVEIKALERDEDADSTTKDRLKLARQKEASLQEELEPLRQRYNEEKHGHEELTQAKKKLDELENKALDAERRYDTATAADLRYFAIPDIKKQIEKLEDQVAEEERRAGANSMIQNVVDSDTISETAARLTGIPVKKLSESENEKLIHMERDLSSEVVGQMDAIKAVSNAVRLSRSGLANPRQPASFLFLGLSGSGKTELAKKVAGFLFNDEDMMIRVDCSELSEKYAVSKLLGTTAGYVGYDEGGFLTNQLQYKPYSVLLFDEVEKAHPDVLTVMLQMLDDGRITSGQGKTIDCSNCIVIMTSNLGAEFINSQQGSKIQESTKNLVMGAVRQHFRPEFLNRISSIVIFNKLSRKAIHKIVDIRLKEIEERFEQNDKHYKLNLTQEAKDFLAKYGYSDDMGARPLNRLIQNEILNKLALRILKNEIKDKETVNVVLKKGKSRDENVPEEAEECLEVLPNHEATIGADTLGDDDNEDSMEIDDDLD |
| CCCS | MSFDTKKATEKVNNVLGEAINLAKEDKHAALTPTHLAVVLFEEPHGLAKVAATKVAGEEVWRSAIRVLRKRLTKLPKVDPAPESVSPGRELSKVLTAAAKLQKDRGDAFLGTDTLLTAVINAAEVSEALGEAGISKAQLETALSEVRQAAGGGPINSETADANFDALAKYGTDLTANAARADPVIGRDDEIRRVVRVLCRRTKNNPVLIGEPGVGKTAIVEGLAQRIVKNDVPETLQGVRLISLDMGSLVAGAKYRGEFEERLKAVLNEVAQQQGKVVLFIDELHLVLGAGKSGDGAMDAANLLKPMLARGELRCIGATTLGEYREHIEKDAAFERRFQQVLVKEPSVPDTIAILRGIKDRYETHHGVHITDRALVVAAELSDRYITTRFLPDKAIDLVDEACANMRVQLDSKPEQLDALERQRQRLQVEAAALAKEKDALSKARAKEVGKELAALEEALRPLQMKYAQEKARLEELRRLGQKRDEILVNIQIAEQHGNLARIADLRYGALPDVEDRIKQVRAAAPSDAMLSEEVGTEEIAVVVSRLTGIPVKKLSESENEKLIHMERDLSSEVVGQMDAIKAVSNAVRLSRSGLANPRQPASFLFLGLSGSGKTELAKKVAGFLFNDEDMMIRVDCSELSEKYAVSKLLGTTAGYVGYDEGGFLTNQLQYKPYSVLLFDEVEKAHPDVLTVMLQMLDDGRITSGQGKTIDCSNCIVIMTSNLGAEFINSQQGSKIQESTKNLVMGAVRQHFRPEFLNRISSIVIFNKLSRKAIHKIVDIRLKEIEERFEQNDKHYKLNLTQEAKDFLAKYGYSDDMGARPLNRLIQNEILNKLALRILKNEIKDKETVNVVLKKGKSRDENVPEEAEECLEVLPNHEATIGADTLGDDDNEDSMEIDDDLD |
| SSCS | MNDQTQFTERALTILTLAQKLASDHQHPQLQPIHILAAFIETPEDGSVPYLQNLIEKGRYDYDLFKKVVNRNLVRIPQQQPAPAEITPSYALGKVLQDAAKIQKQQKDSFIAQDHILFALFNDSSIQQIFKEAQVDIEAIKQQALELRGNTRIDSRGADTNTPLEYLSKYAIDMTEQARQGKLDPVIGREEEIRSTIRVLARRIKSNPCLIGEPGIGKTAIIEGVAQRIIDDDVPTILQGAKLFSLDLAALTAGAKYKGDFEERFKGVLKEIEESKTLIVLFIDEIHMLMGNGKDDAANILKPALSRGQLKVIGATTNNEYRSIVEKDGAFERRFQKIEVAEPSVRQTVAILRGLQPKYEIHHGVRILDSALVTAAQLAKRYLPYRRLPDSALDLVDISCAGVAVARDSKPEQLDALERQRQRLQVEAAALAKEKDALSKARAKEVGKELAALEEALRPLQMKYAQEKARLEELRRLGQKRDEILVNIQIAEQHGNLARIADLRYGALPDVEDRIKQVRAAAPSDAMLSEEVGTEEIAVVVSRLTGIPVKKLSESENEKLIHMERDLSSEVVGQMDAIKAVSNAVRLSRSGLANPRQPASFLFLGLSGSGKTELAKKVAGFLFNDEDMMIRVDCSELSEKYAVSKLLGTTAGYVGYDEGGFLTNQLQYKPYSVLLFDEVEKAHPDVLTVMLQMLDDGRITSGQGKTIDCSNCIVIMTSNLGAEFINSQQGSKIQESTKNLVMGAVRQHFRPEFLNRISSIVIFNKLSRKAIHKIVDIRLKEIEERFEQNDKHYKLNLTQEAKDFLAKYGYSDDMGARPLNRLIQNEILNKLALRILKNEIKDKETVNVVLKKGKSRDENVPEEAEECLEVLPNHEATIGADTLGDDDNEDSMEIDDDLD |
| CSCS | MSFDTKKATEKVNNVLGEAINLAKEDKHAALTPTHLAVVLFEEPHGLAKVAATKVAGEEVWRSAIRVLRKRLTKLPKVDPAPESVSPGRELSKVLTAAAKLQKDRGDAFLGTDTLLTAVINAAEVSEALGEAGISKAQLETALSEVRQARGNTRIDSRGADTNTPLEYLSKYAIDMTEQARQGKLDPVIGREEEIRSTIRVLARRIKSNPCLIGEPGIGKTAIIEGVAQRIIDDDVPTILQGAKLFSLDLAALTAGAKYKGDFEERFKGVLKEIEESKTLIVLFIDEIHMLMGNGKDDAANILKPALSRGQLKVIGATTNNEYRSIVEKDGAFERRFQKIEVAEPSVRQTVAILRGLQPKYEIHHGVRILDSALVTAAQLAKRYLPYRRLPDSALDLVDISCAGVAVARDSKPEQLDALERQRQRLQVEAAALAKEKDALSKARAKEVGKELAALEEALRPLQMKYAQEKARLEELRRLGQKRDEILVNIQIAEQHGNLARIADLRYGALPDVEDRIKQVRAAAPSDAMLSEEVGTEEIAVVVSRLTGIPVKKLSESENEKLIHMERDLSSEVVGQMDAIKAVSNAVRLSRSGLANPRQPASFLFLGLSGSGKTELAKKVAGFLFNDEDMMIRVDCSELSEKYAVSKLLGTTAGYVGYDEGGFLTNQLQYKPYSVLLFDEVEKAHPDVLTVMLQMLDDGRITSGQGKTIDCSNCIVIMTSNLGAEFINSQQGSKIQESTKNLVMGAVRQHFRPEFLNRISSIVIFNKLSRKAIHKIVDIRLKEIEERFEQNDKHYKLNLTQEAKDFLAKYGYSDDMGARPLNRLIQNEILNKLALRILKNEIKDKETVNVVLKKGKSRDENVPEEAEECLEVLPNHEATIGADTLGDDDNEDSMEIDDDLD |
| PSSS | MDPGKFTHKTNEALATAHELTVGAGHAQITPLHLAVALISDPSGIMRQAVANAGDGENTAQAAERVFNQVLKKLPSQSPPPDEVPPSTSLIKVIRRSQALQKSRGDSYLAVDQMILGLLEDSQIRDLFKEVGVSASTVKSEVEKLRGNTRIDSRGADTNTPLEYLSKYAIDMTEQARQGKLDPVIGREEEIRSTIRVLARRIKSNPCLIGEPGIGKTAIIEGVAQRIIDDDVPTILQGAKLFSLDLAALTAGAKYKGDFEERFKGVLKEIEESKTLIVLFIDEIHMLMGNGKDDAANILKPALSRGQLKVIGATTNNEYRSIVEKDGAFERRFQKIEVAEPSVRQTVAILRGLQPKYEIHHGVRILDSALVTAAQLAKRYLPYRRLPDSALDLVDISCAGVAVARDSKPEELDSKERQLQLIQVEIKALERDEDADSTTKDRLKLARQKEASLQEELEPLRQRYNEEKHGHEELTQAKKKLDELENKALDAERRYDTATAADLRYFAIPDIKKQIEKLEDQVAEEERRAGANSMIQNVVDSDTISETAARLTGIPVKKLSESENEKLIHMERDLSSEVVGQMDAIKAVSNAVRLSRSGLANPRQPASFLFLGLSGSGKTELAKKVAGFLFNDEDMMIRVDCSELSEKYAVSKLLGTTAGYVGYDEGGFLTNQLQYKPYSVLLFDEVEKAHPDVLTVMLQMLDDGRITSGQGKTIDCSNCIVIMTSNLGAEFINSQQGSKIQESTKNLVMGAVRQHFRPEFLNRISSIVIFNKLSRKAIHKIVDIRLKEIEERFEQNDKHYKLNLTQEAKDFLAKYGYSDDMGARPLNRLIQNEILNKLALRILKNEIKDKETVNVVLKKGKSRDENVPEEAEECLEVLPNHEATIGADTLGDDDNEDSMEIDDDLD |
| PPSS | MDPGKFTHKTNEALATAHELTVGAGHAQITPLHLAVALISDPSGIMRQAVANAGDGENTAQAAERVFNQVLKKLPSQSPPPDEVPPSTSLIKVIRRSQALQKSRGDSYLAVDQMILGLLEDSQIRDLFKEVGVSASTVKSEVEKLRGKEGKKVENASGDTNFQALKTYGRDLVEGAGKLDPVIGRDEEIRRVVRILSRRTKNNPVLIGEPGVGKTAVAEGLAQRIVRGDVPSNLADVRLIALDMGALVAGAKYRGEFEERLKAVLKEVEEAEGKVILFIDEIHLVLGAGRTEGSMDAANLFKPMLARGQLRCIGATTLEEYRKYVEKDAAFERRFQQVYVAEPSVPDTISILRGLKEKYEGHHGVRIQDRALVIAAQLSSRYITGRHLPDKAIDLVDEACANVRVQLDSKPEELDSKERQLQLIQVEIKALERDEDADSTTKDRLKLARQKEASLQEELEPLRQRYNEEKHGHEELTQAKKKLDELENKALDAERRYDTATAADLRYFAIPDIKKQIEKLEDQVAEEERRAGANSMIQNVVDSDTISETAARLTGIPVKKLSESENEKLIHMERDLSSEVVGQMDAIKAVSNAVRLSRSGLANPRQPASFLFLGLSGSGKTELAKKVAGFLFNDEDMMIRVDCSELSEKYAVSKLLGTTAGYVGYDEGGFLTNQLQYKPYSVLLFDEVEKAHPDVLTVMLQMLDDGRITSGQGKTIDCSNCIVIMTSNLGAEFINSQQGSKIQESTKNLVMGAVRQHFRPEFLNRISSIVIFNKLSRKAIHKIVDIRLKEIEERFEQNDKHYKLNLTQEAKDFLAKYGYSDDMGARPLNRLIQNEILNKLALRILKNEIKDKETVNVVLKKGKSRDENVPEEAEECLEVLPNHEATIGADTLGDDDNEDSMEIDDDLD |
| PPPS | MDPGKFTHKTNEALATAHELTVGAGHAQITPLHLAVALISDPSGIMRQAVANAGDGENTAQAAERVFNQVLKKLPSQSPPPDEVPPSTSLIKVIRRSQALQKSRGDSYLAVDQMILGLLEDSQIRDLFKEVGVSASTVKSEVEKLRGKEGKKVENASGDTNFQALKTYGRDLVEGAGKLDPVIGRDEEIRRVVRILSRRTKNNPVLIGEPGVGKTAVAEGLAQRIVRGDVPSNLADVRLIALDMGALVAGAKYRGEFEERLKAVLKEVEEAEGKVILFIDEIHLVLGAGRTEGSMDAANLFKPMLARGQLRCIGATTLEEYRKYVEKDAAFERRFQQVYVAEPSVPDTISILRGLKEKYEGHHGVRIQDRALVIAAQLSSRYITGRHLPDKAIDLVDEACANVRVQLDSQPEEIDSLERKRMQLEVELHALEKEKDKASKARLAEVVKELDDLRDKLQPLLMKYKKEKERIDEIRRLKQKREEILFSIQEAERRYDLARVADLRYGALEEVEAAIARLEGSTTDENLMLTETVGPEHIAEVVSRLTGIPVKKLSESENEKLIHMERDLSSEVVGQMDAIKAVSNAVRLSRSGLANPRQPASFLFLGLSGSGKTELAKKVAGFLFNDEDMMIRVDCSELSEKYAVSKLLGTTAGYVGYDEGGFLTNQLQYKPYSVLLFDEVEKAHPDVLTVMLQMLDDGRITSGQGKTIDCSNCIVIMTSNLGAEFINSQQGSKIQESTKNLVMGAVRQHFRPEFLNRISSIVIFNKLSRKAIHKIVDIRLKEIEERFEQNDKHYKLNLTQEAKDFLAKYGYSDDMGARPLNRLIQNEILNKLALRILKNEIKDKETVNVVLKKGKSRDENVPEEAEECLEVLPNHEATIGADTLGDDDNEDSMEIDDDLD |
| SSPS | MNDQTQFTERALTILTLAQKLASDHQHPQLQPIHILAAFIETPEDGSVPYLQNLIEKGRYDYDLFKKVVNRNLVRIPQQQPAPAEITPSYALGKVLQDAAKIQKQQKDSFIAQDHILFALFNDSSIQQIFKEAQVDIEAIKQQALELRGNTRIDSRGADTNTPLEYLSKYAIDMTEQARQGKLDPVIGREEEIRSTIRVLARRIKSNPCLIGEPGIGKTAIIEGVAQRIIDDDVPTILQGAKLFSLDLAALTAGAKYKGDFEERFKGVLKEIEESKTLIVLFIDEIHMLMGNGKDDAANILKPALSRGQLKVIGATTNNEYRSIVEKDGAFERRFQKIEVAEPSVRQTVAILRGLQPKYEIHHGVRILDSALVTAAQLAKRYLPYRRLPDSALDLVDISCAGVAVARDSQPEEIDSLERKRMQLEVELHALEKEKDKASKARLAEVVKELDDLRDKLQPLLMKYKKEKERIDEIRRLKQKREEILFSIQEAERRYDLARVADLRYGALEEVEAAIARLEGSTTDENLMLTETVGPEHIAEVVSRLTGIPVKKLSESENEKLIHMERDLSSEVVGQMDAIKAVSNAVRLSRSGLANPRQPASFLFLGLSGSGKTELAKKVAGFLFNDEDMMIRVDCSELSEKYAVSKLLGTTAGYVGYDEGGFLTNQLQYKPYSVLLFDEVEKAHPDVLTVMLQMLDDGRITSGQGKTIDCSNCIVIMTSNLGAEFINSQQGSKIQESTKNLVMGAVRQHFRPEFLNRISSIVIFNKLSRKAIHKIVDIRLKEIEERFEQNDKHYKLNLTQEAKDFLAKYGYSDDMGARPLNRLIQNEILNKLALRILKNEIKDKETVNVVLKKGKSRDENVPEEAEECLEVLPNHEATIGADTLGDDDNEDSMEIDDDLD |
| PSPS | MDPGKFTHKTNEALATAHELTVGAGHAQITPLHLAVALISDPSGIMRQAVANAGDGENTAQAAERVFNQVLKKLPSQSPPPDEVPPSTSLIKVIRRSQALQKSRGDSYLAVDQMILGLLEDSQIRDLFKEVGVSASTVKSEVEKLRGNTRIDSRGADTNTPLEYLSKYAIDMTEQARQGKLDPVIGREEEIRSTIRVLARRIKSNPCLIGEPGIGKTAIIEGVAQRIIDDDVPTILQGAKLFSLDLAALTAGAKYKGDFEERFKGVLKEIEESKTLIVLFIDEIHMLMGNGKDDAANILKPALSRGQLKVIGATTNNEYRSIVEKDGAFERRFQKIEVAEPSVRQTVAILRGLQPKYEIHHGVRILDSALVTAAQLAKRYLPYRRLPDSALDLVDISCAGVAVARDSQPEEIDSLERKRMQLEVELHALEKEKDKASKARLAEVVKELDDLRDKLQPLLMKYKKEKERIDEIRRLKQKREEILFSIQEAERRYDLARVADLRYGALEEVEAAIARLEGSTTDENLMLTETVGPEHIAEVVSRLTGIPVKKLSESENEKLIHMERDLSSEVVGQMDAIKAVSNAVRLSRSGLANPRQPASFLFLGLSGSGKTELAKKVAGFLFNDEDMMIRVDCSELSEKYAVSKLLGTTAGYVGYDEGGFLTNQLQYKPYSVLLFDEVEKAHPDVLTVMLQMLDDGRITSGQGKTIDCSNCIVIMTSNLGAEFINSQQGSKIQESTKNLVMGAVRQHFRPEFLNRISSIVIFNKLSRKAIHKIVDIRLKEIEERFEQNDKHYKLNLTQEAKDFLAKYGYSDDMGARPLNRLIQNEILNKLALRILKNEIKDKETVNVVLKKGKSRDENVPEEAEECLEVLPNHEATIGADTLGDDDNEDSMEIDDDLD |
| RSSS | MALNPNEWTEKVQEMYLEAKNVAINNKNAYMDPIHFAVALFEDEGGLPQRVVQKSGASLDAVEGAMRSLLKAIPQQDPAPVDVSTSHKALRFLQNAQKKQKKNDEAHLAIDHLLLALVQEKDILQALAGCGLAKDRFEEIVKKIRGNTRIDSRGADTNTPLEYLSKYAIDMTEQARQGKLDPVIGREEEIRSTIRVLARRIKSNPCLIGEPGIGKTAIIEGVAQRIIDDDVPTILQGAKLFSLDLAALTAGAKYKGDFEERFKGVLKEIEESKTLIVLFIDEIHMLMGNGKDDAANILKPALSRGQLKVIGATTNNEYRSIVEKDGAFERRFQKIEVAEPSVRQTVAILRGLQPKYEIHHGVRILDSALVTAAQLAKRYLPYRRLPDSALDLVDISCAGVAVARDSKPEELDSKERQLQLIQVEIKALERDEDADSTTKDRLKLARQKEASLQEELEPLRQRYNEEKHGHEELTQAKKKLDELENKALDAERRYDTATAADLRYFAIPDIKKQIEKLEDQVAEEERRAGANSMIQNVVDSDTISETAARLTGIPVKKLSESENEKLIHMERDLSSEVVGQMDAIKAVSNAVRLSRSGLANPRQPASFLFLGLSGSGKTELAKKVAGFLFNDEDMMIRVDCSELSEKYAVSKLLGTTAGYVGYDEGGFLTNQLQYKPYSVLLFDEVEKAHPDVLTVMLQMLDDGRITSGQGKTIDCSNCIVIMTSNLGAEFINSQQGSKIQESTKNLVMGAVRQHFRPEFLNRISSIVIFNKLSRKAIHKIVDIRLKEIEERFEQNDKHYKLNLTQEAKDFLAKYGYSDDMGARPLNRLIQNEILNKLALRILKNEIKDKETVNVVLKKGKSRDENVPEEAEECLEVLPNHEATIGADTLGDDDNEDSMEIDDDLD |
| RRSS | MALNPNEWTEKVQEMYLEAKNVAINNKNAYMDPIHFAVALFEDEGGLPQRVVQKSGASLDAVEGAMRSLLKAIPQQDPAPVDVSTSHKALRFLQNAQKKQKKNDEAHLAIDHLLLALVQEKDILQALAGCGLAKDRFEEIVKKIKGTTRANTKTAESTYDALGKYGVDLVQRAADGKLDPVIGRDEEIRRVIQILARRTKNNPVLVGPPGTGKTAIVEGLAQRILNGDVPETLKARLVSLDMGALIAGAKYRGEFEERLKSVLDEVKQAEGSIILFVDEIHTVLGAGKTEGSMDAANLLKPMLARGELRMIGATTLDEYRKHVEKDAAFERRFQMVHVSEPSVPDTVSILRGLKERYEAHHGVRIQDAALVTAAQLADRYITQRFLPDKAIDLVDEACAKTRVQLDSKPEELDSKERQLQLIQVEIKALERDEDADSTTKDRLKLARQKEASLQEELEPLRQRYNEEKHGHEELTQAKKKLDELENKALDAERRYDTATAADLRYFAIPDIKKQIEKLEDQVAEEERRAGANSMIQNVVDSDTISETAARLTGIPVKKLSESENEKLIHMERDLSSEVVGQMDAIKAVSNAVRLSRSGLANPRQPASFLFLGLSGSGKTELAKKVAGFLFNDEDMMIRVDCSELSEKYAVSKLLGTTAGYVGYDEGGFLTNQLQYKPYSVLLFDEVEKAHPDVLTVMLQMLDDGRITSGQGKTIDCSNCIVIMTSNLGAEFINSQQGSKIQESTKNLVMGAVRQHFRPEFLNRISSIVIFNKLSRKAIHKIVDIRLKEIEERFEQNDKHYKLNLTQEAKDFLAKYGYSDDMGARPLNRLIQNEILNKLALRILKNEIKDKETVNVVLKKGKSRDENVPEEAEECLEVLPNHEATIGADTLGDDDNEDSMEIDDDLD |
| RRRS | MALNPNEWTEKVQEMYLEAKNVAINNKNAYMDPIHFAVALFEDEGGLPQRVVQKSGASLDAVEGAMRSLLKAIPQQDPAPVDVSTSHKALRFLQNAQKKQKKNDEAHLAIDHLLLALVQEKDILQALAGCGLAKDRFEEIVKKIKGTTRANTKTAESTYDALGKYGVDLVQRAADGKLDPVIGRDEEIRRVIQILARRTKNNPVLVGPPGTGKTAIVEGLAQRILNGDVPETLKARLVSLDMGALIAGAKYRGEFEERLKSVLDEVKQAEGSIILFVDEIHTVLGAGKTEGSMDAANLLKPMLARGELRMIGATTLDEYRKHVEKDAAFERRFQMVHVSEPSVPDTVSILRGLKERYEAHHGVRIQDAALVTAAQLADRYITQRFLPDKAIDLVDEACAKTRVQLDSRPEEIDALERRKLQLEVEATALGKEKDKMSKQRLKEVKKQLADIEEQLGPLKMKFEMERGRVDEMRELQEKLDNLRNKVQRAERAGDLSTAADLKYYAIPDCEKRLKQLIEEQEKRQQEQQSMDVSDEDKPMLSEEVGPDQVTEIVARLTGIPVKKLSESENEKLIHMERDLSSEVVGQMDAIKAVSNAVRLSRSGLANPRQPASFLFLGLSGSGKTELAKKVAGFLFNDEDMMIRVDCSELSEKYAVSKLLGTTAGYVGYDEGGFLTNQLQYKPYSVLLFDEVEKAHPDVLTVMLQMLDDGRITSGQGKTIDCSNCIVIMTSNLGAEFINSQQGSKIQESTKNLVMGAVRQHFRPEFLNRISSIVIFNKLSRKAIHKIVDIRLKEIEERFEQNDKHYKLNLTQEAKDFLAKYGYSDDMGARPLNRLIQNEILNKLALRILKNEIKDKETVNVVLKKGKSRDENVPEEAEECLEVLPNHEATIGADTLGDDDNEDSMEIDDDLD |
| SSRS | MNDQTQFTERALTILTLAQKLASDHQHPQLQPIHILAAFIETPEDGSVPYLQNLIEKGRYDYDLFKKVVNRNLVRIPQQQPAPAEITPSYALGKVLQDAAKIQKQQKDSFIAQDHILFALFNDSSIQQIFKEAQVDIEAIKQQALELRGNTRIDSRGADTNTPLEYLSKYAIDMTEQARQGKLDPVIGREEEIRSTIRVLARRIKSNPCLIGEPGIGKTAIIEGVAQRIIDDDVPTILQGAKLFSLDLAALTAGAKYKGDFEERFKGVLKEIEESKTLIVLFIDEIHMLMGNGKDDAANILKPALSRGQLKVIGATTNNEYRSIVEKDGAFERRFQKIEVAEPSVRQTVAILRGLQPKYEIHHGVRILDSALVTAAQLAKRYLPYRRLPDSALDLVDISCAGVAVARDSRPEEIDALERRKLQLEVEATALGKEKDKMSKQRLKEVKKQLADIEEQLGPLKMKFEMERGRVDEMRELQEKLDNLRNKVQRAERAGDLSTAADLKYYAIPDCEKRLKQLIEEQEKRQQEQQSMDVSDEDKPMLSEEVGPDQVTEIVARLTGIPVKKLSESENEKLIHMERDLSSEVVGQMDAIKAVSNAVRLSRSGLANPRQPASFLFLGLSGSGKTELAKKVAGFLFNDEDMMIRVDCSELSEKYAVSKLLGTTAGYVGYDEGGFLTNQLQYKPYSVLLFDEVEKAHPDVLTVMLQMLDDGRITSGQGKTIDCSNCIVIMTSNLGAEFINSQQGSKIQESTKNLVMGAVRQHFRPEFLNRISSIVIFNKLSRKAIHKIVDIRLKEIEERFEQNDKHYKLNLTQEAKDFLAKYGYSDDMGARPLNRLIQNEILNKLALRILKNEIKDKETVNVVLKKGKSRDENVPEEAEECLEVLPNHEATIGADTLGDDDNEDSMEIDDDLD |
| RSRS | MALNPNEWTEKVQEMYLEAKNVAINNKNAYMDPIHFAVALFEDEGGLPQRVVQKSGASLDAVEGAMRSLLKAIPQQDPAPVDVSTSHKALRFLQNAQKKQKKNDEAHLAIDHLLLALVQEKDILQALAGCGLAKDRFEEIVKKIRGNTRIDSRGADTNTPLEYLSKYAIDMTEQARQGKLDPVIGREEEIRSTIRVLARRIKSNPCLIGEPGIGKTAIIEGVAQRIIDDDVPTILQGAKLFSLDLAALTAGAKYKGDFEERFKGVLKEIEESKTLIVLFIDEIHMLMGNGKDDAANILKPALSRGQLKVIGATTNNEYRSIVEKDGAFERRFQKIEVAEPSVRQTVAILRGLQPKYEIHHGVRILDSALVTAAQLAKRYLPYRRLPDSALDLVDISCAGVAVARDSRPEEIDALERRKLQLEVEATALGKEKDKMSKQRLKEVKKQLADIEEQLGPLKMKFEMERGRVDEMRELQEKLDNLRNKVQRAERAGDLSTAADLKYYAIPDCEKRLKQLIEEQEKRQQEQQSMDVSDEDKPMLSEEVGPDQVTEIVARLTGIPVKKLSESENEKLIHMERDLSSEVVGQMDAIKAVSNAVRLSRSGLANPRQPASFLFLGLSGSGKTELAKKVAGFLFNDEDMMIRVDCSELSEKYAVSKLLGTTAGYVGYDEGGFLTNQLQYKPYSVLLFDEVEKAHPDVLTVMLQMLDDGRITSGQGKTIDCSNCIVIMTSNLGAEFINSQQGSKIQESTKNLVMGAVRQHFRPEFLNRISSIVIFNKLSRKAIHKIVDIRLKEIEERFEQNDKHYKLNLTQEAKDFLAKYGYSDDMGARPLNRLIQNEILNKLALRILKNEIKDKETVNVVLKKGKSRDENVPEEAEECLEVLPNHEATIGADTLGDDDNEDSMEIDDDLD |
| CaSSS | MSSMQFTDKATETLNAAAKYAAENSHVQLHPSHVAVVMLDEENSLFRSILEKAGGDVVSIERGFKKIMVRQPSQDPPPTEMGHSPELAKLLHYAHEHMKKQRDLYIAQDHLILALADLPSMAQVLKEGGVTKKSLENAVTHVRGNTRIDSRGADTNTPLEYLSKYAIDMTEQARQGKLDPVIGREEEIRSTIRVLARRIKSNPCLIGEPGIGKTAIIEGVAQRIIDDDVPTILQGAKLFSLDLAALTAGAKYKGDFEERFKGVLKEIEESKTLIVLFIDEIHMLMGNGKDDAANILKPALSRGQLKVIGATTNNEYRSIVEKDGAFERRFQKIEVAEPSVRQTVAILRGLQPKYEIHHGVRILDSALVTAAQLAKRYLPYRRLPDSALDLVDISCAGVAVARDSKPEELDSKERQLQLIQVEIKALERDEDADSTTKDRLKLARQKEASLQEELEPLRQRYNEEKHGHEELTQAKKKLDELENKALDAERRYDTATAADLRYFAIPDIKKQIEKLEDQVAEEERRAGANSMIQNVVDSDTISETAARLTGIPVKKLSESENEKLIHMERDLSSEVVGQMDAIKAVSNAVRLSRSGLANPRQPASFLFLGLSGSGKTELAKKVAGFLFNDEDMMIRVDCSELSEKYAVSKLLGTTAGYVGYDEGGFLTNQLQYKPYSVLLFDEVEKAHPDVLTVMLQMLDDGRITSGQGKTIDCSNCIVIMTSNLGAEFINSQQGSKIQESTKNLVMGAVRQHFRPEFLNRISSIVIFNKLSRKAIHKIVDIRLKEIEERFEQNDKHYKLNLTQEAKDFLAKYGYSDDMGARPLNRLIQNEILNKLALRILKNEIKDKETVNVVLKKGKSRDENVPEEAEECLEVLPNHEATIGADTLGDDDNEDSMEIDDDLD |
| CaCaSS | MSSMQFTDKATETLNAAAKYAAENSHVQLHPSHVAVVMLDEENSLFRSILEKAGGDVVSIERGFKKIMVRQPSQDPPPTEMGHSPELAKLLHYAHEHMKKQRDLYIAQDHLILALADLPSMAQVLKEGGVTKKSLENAVTHVRGNRRVESKSAEEAYEALSKYCIDLTELAASGKLDPVIGRDEIISRVIRVLSRRTKNNPCLVGEPGVGKTAIAEGLANRIVKGDIPSSLQKKVYSLDIGSLLAGAKYRGEFEERLKAVLKELKEAQAIVFIDEIHTVLGAGKSEGAIDAANLLKPMLARGELRCIGATTLTEYRQYVEKDPAFERRFQLVMVEEPSVTDTISILRGLKERYETHHGVRIADAAIVAAAQLAARYITQRFMPDKAIDLIDEACANTRVQLDSKPEELDSKERQLQLIQVEIKALERDEDADSTTKDRLKLARQKEASLQEELEPLRQRYNEEKHGHEELTQAKKKLDELENKALDAERRYDTATAADLRYFAIPDIKKQIEKLEDQVAEEERRAGANSMIQNVVDSDTISETAARLTGIPVKKLSESENEKLIHMERDLSSEVVGQMDAIKAVSNAVRLSRSGLANPRQPASFLFLGLSGSGKTELAKKVAGFLFNDEDMMIRVDCSELSEKYAVSKLLGTTAGYVGYDEGGFLTNQLQYKPYSVLLFDEVEKAHPDVLTVMLQMLDDGRITSGQGKTIDCSNCIVIMTSNLGAEFINSQQGSKIQESTKNLVMGAVRQHFRPEFLNRISSIVIFNKLSRKAIHKIVDIRLKEIEERFEQNDKHYKLNLTQEAKDFLAKYGYSDDMGARPLNRLIQNEILNKLALRILKNEIKDKETVNVVLKKGKSRDENVPEEAEECLEVLPNHEATIGADTLGDDDNEDSMEIDDDLD |
| CaCaCaS | MSSMQFTDKATETLNAAAKYAAENSHVQLHPSHVAVVMLDEENSLFRSILEKAGGDVVSIERGFKKIMVRQPSQDPPPTEMGHSPELAKLLHYAHEHMKKQRDLYIAQDHLILALADLPSMAQVLKEGGVTKKSLENAVTHVRGNRRVESKSAEEAYEALSKYCIDLTELAASGKLDPVIGRDEIISRVIRVLSRRTKNNPCLVGEPGVGKTAIAEGLANRIVKGDIPSSLQKKVYSLDIGSLLAGAKYRGEFEERLKAVLKELKEAQAIVFIDEIHTVLGAGKSEGAIDAANLLKPMLARGELRCIGATTLTEYRQYVEKDPAFERRFQLVMVEEPSVTDTISILRGLKERYETHHGVRIADAAIVAAAQLAARYITQRFMPDKAIDLIDEACANTRVQLDSQPEAIDKLERRHLQLEVEATALEKEKDAASKQRLQEVRAEMARIQEELRPLKMKYESEKGRLDEIRNLSQRLDELKAKAEDAERRYDLARAADIRYYAIPDLEKRLAQLQAEKSQADAERADGLLAEVVGPDQIMEVVSRLTGIPVKKLSESENEKLIHMERDLSSEVVGQMDAIKAVSNAVRLSRSGLANPRQPASFLFLGLSGSGKTELAKKVAGFLFNDEDMMIRVDCSELSEKYAVSKLLGTTAGYVGYDEGGFLTNQLQYKPYSVLLFDEVEKAHPDVLTVMLQMLDDGRITSGQGKTIDCSNCIVIMTSNLGAEFINSQQGSKIQESTKNLVMGAVRQHFRPEFLNRISSIVIFNKLSRKAIHKIVDIRLKEIEERFEQNDKHYKLNLTQEAKDFLAKYGYSDDMGARPLNRLIQNEILNKLALRILKNEIKDKETVNVVLKKGKSRDENVPEEAEECLEVLPNHEATIGADTLGDDDNEDSMEIDDDLD |
| SSCaS | MNDQTQFTERALTILTLAQKLASDHQHPQLQPIHILAAFIETPEDGSVPYLQNLIEKGRYDYDLFKKVVNRNLVRIPQQQPAPAEITPSYALGKVLQDAAKIQKQQKDSFIAQDHILFALFNDSSIQQIFKEAQVDIEAIKQQALELRGNTRIDSRGADTNTPLEYLSKYAIDMTEQARQGKLDPVIGREEEIRSTIRVLARRIKSNPCLIGEPGIGKTAIIEGVAQRIIDDDVPTILQGAKLFSLDLAALTAGAKYKGDFEERFKGVLKEIEESKTLIVLFIDEIHMLMGNGKDDAANILKPALSRGQLKVIGATTNNEYRSIVEKDGAFERRFQKIEVAEPSVRQTVAILRGLQPKYEIHHGVRILDSALVTAAQLAKRYLPYRRLPDSALDLVDISCAGVAVARDSQPEAIDKLERRHLQLEVEATALEKEKDAASKQRLQEVRAEMARIQEELRPLKMKYESEKGRLDEIRNLSQRLDELKAKAEDAERRYDLARAADIRYYAIPDLEKRLAQLQAEKSQADAERADGLLAEVVGPDQIMEVVSRLTGIPVKKLSESENEKLIHMERDLSSEVVGQMDAIKAVSNAVRLSRSGLANPRQPASFLFLGLSGSGKTELAKKVAGFLFNDEDMMIRVDCSELSEKYAVSKLLGTTAGYVGYDEGGFLTNQLQYKPYSVLLFDEVEKAHPDVLTVMLQMLDDGRITSGQGKTIDCSNCIVIMTSNLGAEFINSQQGSKIQESTKNLVMGAVRQHFRPEFLNRISSIVIFNKLSRKAIHKIVDIRLKEIEERFEQNDKHYKLNLTQEAKDFLAKYGYSDDMGARPLNRLIQNEILNKLALRILKNEIKDKETVNVVLKKGKSRDENVPEEAEECLEVLPNHEATIGADTLGDDDNEDSMEIDDDLD |
| CaSCaS | MSSMQFTDKATETLNAAAKYAAENSHVQLHPSHVAVVMLDEENSLFRSILEKAGGDVVSIERGFKKIMVRQPSQDPPPTEMGHSPELAKLLHYAHEHMKKQRDLYIAQDHLILALADLPSMAQVLKEGGVTKKSLENAVTHVRGNTRIDSRGADTNTPLEYLSKYAIDMTEQARQGKLDPVIGREEEIRSTIRVLARRIKSNPCLIGEPGIGKTAIIEGVAQRIIDDDVPTILQGAKLFSLDLAALTAGAKYKGDFEERFKGVLKEIEESKTLIVLFIDEIHMLMGNGKDDAANILKPALSRGQLKVIGATTNNEYRSIVEKDGAFERRFQKIEVAEPSVRQTVAILRGLQPKYEIHHGVRILDSALVTAAQLAKRYLPYRRLPDSALDLVDISCAGVAVARDSQPEAIDKLERRHLQLEVEATALEKEKDAASKQRLQEVRAEMARIQEELRPLKMKYESEKGRLDEIRNLSQRLDELKAKAEDAERRYDLARAADIRYYAIPDLEKRLAQLQAEKSQADAERADGLLAEVVGPDQIMEVVSRLTGIPVKKLSESENEKLIHMERDLSSEVVGQMDAIKAVSNAVRLSRSGLANPRQPASFLFLGLSGSGKTELAKKVAGFLFNDEDMMIRVDCSELSEKYAVSKLLGTTAGYVGYDEGGFLTNQLQYKPYSVLLFDEVEKAHPDVLTVMLQMLDDGRITSGQGKTIDCSNCIVIMTSNLGAEFINSQQGSKIQESTKNLVMGAVRQHFRPEFLNRISSIVIFNKLSRKAIHKIVDIRLKEIEERFEQNDKHYKLNLTQEAKDFLAKYGYSDDMGARPLNRLIQNEILNKLALRILKNEIKDKETVNVVLKKGKSRDENVPEEAEECLEVLPNHEATIGADTLGDDDNEDSMEIDDDLD |
